# Tissue-specific progesterone receptor-chromatin binding and the regulation of progesterone-dependent gene expression

**DOI:** 10.1038/s41598-019-48333-8

**Published:** 2019-08-19

**Authors:** D. T. Dinh, J. Breen, L. K. Akison, F. J. DeMayo, H. M. Brown, R. L. Robker, D. L. Russell

**Affiliations:** 1 Robinson Research Institute, Adelaide Medical School, University of Adelaide, Adelaide, Australia; 2 University of Adelaide Bioinformatics Hub, University of Adelaide, Adelaide, Australia; 3 Child Health Research Centre, Centre for Children’s Health Research, The University of Queensland, South Brisbane, Qld 4101 Australia; 4 Pregnancy and Female Reproduction Group, National Institute of Environmental Health Sciences, Research Triangle Park, Durham, NC 27709 USA; 5 South Australian Health and Medical Research Institute, Adelaide, South Australia Australia; 6 Australian Research Council (ARC) Centre for Nanoscale Biophotonics, University of Adelaide, Adelaide, Australia

**Keywords:** Hormone receptors, Transcriptional regulatory elements, Ovary

## Abstract

Progesterone receptor (PGR) co-ordinately regulates ovulation, fertilisation and embryo implantation through tissue-specific actions, but the mechanisms for divergent PGR action are poorly understood. Here we characterised PGR activity in mouse granulosa cells using combined ChIP-seq for PGR and H3K27ac and gene expression microarray. Comparison of granulosa, uterus and oviduct PGR-dependent genes showed almost complete tissue specificity in PGR target gene profiles. In granulosa cells 82% of identified PGR-regulated genes bound PGR within 3 kb of the gene and PGR binding sites were highly enriched in proximal promoter regions in close proximity to H3K27ac-modified active chromatin. Motif analysis showed highly enriched PGR binding to the PGR response element (GnACAnnnTGTnC), but PGR also interacted significantly with other transcription factor binding motifs. In uterus PGR showed far more tendency to bind intergenic chromatin regions and low evidence of interaction with other transcription factors. This is the first genome-wide description of PGR action in granulosa cells and systematic comparison of diverse PGR action in different reproductive tissues. It clarifies finely-tuned contextual PGR-chromatin interactions with implications for more targeted reproductive medicine.

## Introduction

Progesterone (P4) is an essential reproductive hormone produced by the ovarian follicular granulosa cells immediately prior to ovulation. P4 has diverse pleiotropic roles^1–4^, including endometrial differentiation prior to implantation^5^ and mammary epithelial cell proliferation and differentiation^6,7^. In ovarian follicles, P4 plays a primary role in controlling fertility as the essential mediator of ovulation^4,8,9^. P4 mainly functions through the direct binding and activation of progesterone receptor (PGR), a nuclear receptor that has profound importance in the regulation and maintenance of normal reproductive physiology. PGR belongs to the 3-Ketosteroid receptor family (NR3C) and there are two isoforms of PGR, PGR-A and PGR-B, which are identical apart from an additional N-terminal domain in PGR-B^10^. Both isoforms are present in most PGR-positive cells; however, PGR-A is more important for ovarian and uterine functions whereas PGR-B plays the main role in the murine mammary gland^11,12^.

In reproductive tissues, PGR shows distinct functions that are highly dependent on the tissue context, revealed in studies on PGR knockout (PRKO) mouse models^13^. In the pre-ovulatory ovary, PGR is expressed exclusively in granulosa cells in response to the ovulatory LH-surge^14^. PRKO female mice are infertile due to complete anovulation^13^, although their oocytes are capable of developing into normal pups^15^. The ovulatory role of PGR is also critical in primates and humans as illustrated by PGR antagonist or gene knockdown^16^. In the ovary, PGR is responsible for the induction of genes that are critical for ovulation, such as *Adamts1* in granulosa cells^17–19^. PGR also plays a number of roles in the reproductive tract, such as regulating inflammation in the ovaries, ciliated transporting of embryos and promoting decidualisation and implantation in the uterus^13,20,21^. These are achieved through the regulation of target genes; such as *Itga8* and *Edn3* in the oviduct^20^, and *Cited2* and *Gata2* in the uterus^22,23^. Although PGR regulates large suites of genes in many reproductive tissues, the modulating effect of PGR is highly tissue-specific. However, most studies on PGR-dependent transcriptome profile have been performed independently and there has been no direct comparative investigation across different target tissues.

The canonical PGR-dependent transcriptional regulation is most well-studied in breast cancer in which PGR is a ligand-dependent nuclear transcription factor (TF)^24^. Upon binding P4, activated PGR translocates into the nucleus and binds to regulatory motifs, most often containing a PGR response element (PRE). The canonical PRE is an inverted palindrome (5′-ACAnnnTGT-3′), but it is recognised that the PRE motif can vary depending on neighbouring TF binding or other chromatin modifiers^25^. The influence of PGR is also not uniquely restricted to genes with known PRE, as interaction between PGR and other TF can recruit PGR to non-consensus motifs^26,27^. This has been reported in the ovary for PGR-induced *Adamts1* which does not have PRE in the defined regulatory region^28^. *Adamts1* possesses G/C-rich regions in the proximal promoter region that bind to SP1/ SP3 co-mediators and an interaction between PGR and SP1/ SP3 at these sites has been proposed as a mechanism for PGR-mediated gene regulation^28^.

Studies to date on PGR action have largely focused on identifying PGR-regulated genes using targeted reporter assays or genomic screening^28–30^, which does little to explain the selective and tissue-dependent action of PGR on the genome. Recent studies have begun to define the molecular pathway of PGR action in the reproductive tract, assisted by improved genome-wide molecular technologies^22,31,32^. However, no studies have yet investigated the molecular pathway involving PGR in the ovary or how the specialised physiological roles in different reproductive organs are achieved through the same receptor signalling mechanism. An understanding of the mechanism responsible for the diversity in PGR action between different target tissues may reveal key details of PGR functions; considering how differently PGR behaves between cell types, including normal versus cancerous cells, it is valuable to actively investigate these contrasting regulatory mechanisms.

In this study, we investigated the molecular mechanism of PGR in the ovary and combined our findings with data from similar studies to investigate tissue specificity in action. Using microarray analysis of PRKO granulosa cells, we identified the PGR-dependent transcriptome during the peri-ovulatory period. We defined the PGR chromatin-binding cistrome in peri-ovulatory granulosa cells using ChIP-seq and combined these two datasets to characterise PGR interaction with its target genes in granulosa cells. Comparison of the PGR-dependent transcriptome in granulosa cells with that in the uterus and oviduct showed the extremely diverse response of PGR in these distinct but highly related target organs. Furthermore, comparison of the PGR-binding cistrome in granulosa and endometrial cells indicated that PGR tissue specificity might be driven by interaction with specific and unique accessory TF.

## Results

### Quantification of PGR expression in granulosa cells

**Figure 1:**
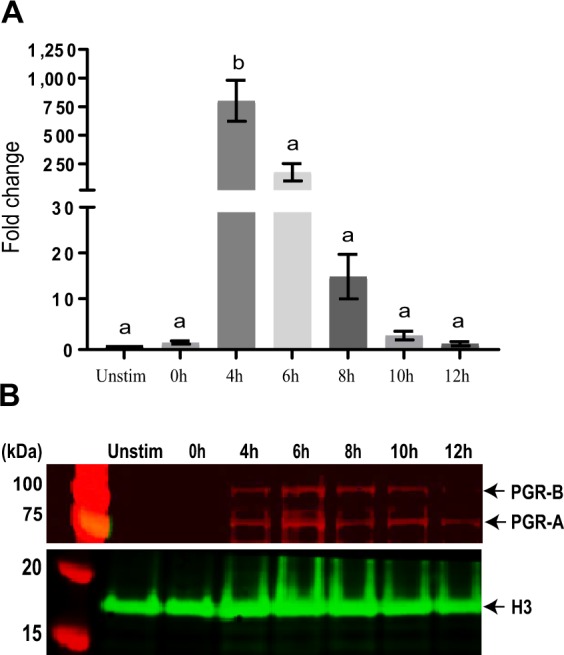
PGR mRNA and protein are induced by the LH surge in granulosa cells. (**A**) PGR mRNA expression in eCG and hCG-primed granulosa cells at no eCG + hCG (unstim) and eCG + hCG 0–12 h post-hCG stimulation. RT-qPCR was performed on samples of 1–3 ovaries. N = 3 independent experiments, bars with different superscripts are significantly different, F_6,14_ = 17.53, p < 0.0001 (one-way ANOVA). (**B**) Western blot of PGR in granulosa cells during ovulation (unstim or eCG + hCG 0–12 h post-hCG). Western blot was performed in biological triplicates, with 3–4 mice per time point in each replicate. One representative example of three highly similar results is shown. Full-length blot is available in Suppl. Data 3.

To confirm the temporal pattern of *Pgr* expression during ovulation, we performed RT-qPCR on mouse granulosa cells at various human chorionic gonadotropin (hCG)-stimulated time points. The expression of *Pgr* was significantly and transiently induced during the pre-ovulatory period, peaking at 4 h post-hCG stimulation (Fig. 1A). Western blot on protein extracts across the same time-course confirmed the mRNA observation and showed both isoforms – PGR-A (83 kDa) and PGR-B (115 kDa) – induced from 4 h post-hCG, achieving highest intensity at the 6 h time point (Fig. 1B). As PGR protein level was highest at 6 h post-hCG, we chose this time point to collect our samples for subsequent experiments.

### Characterisation of PGR and H3K27ac binding in granulosa cell genome

**Figure 2:**
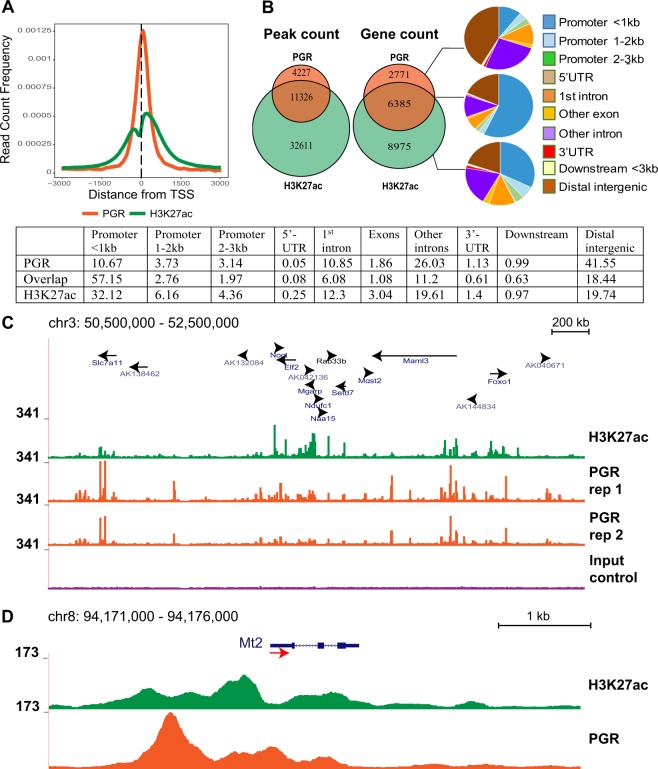
PGR induced by ovulatory LH surge rapidly associates with active enhancers throughout the granulosa cell genome. (**A**) Read count frequency of PGR peaks (orange) and H3K27ac peaks (green) in relation to TSS. (**B**) Venn diagrams showing the peak count for PGR and H3K27ac (left) and gene with peaks (right). Peaks were divided into PGR-unique, overlapped and H3K27ac-unique sections and analysed for genome distribution. The promoter region was defined as 3 kb upstream of TSS and divided into three sections: <1 kb, 1–2 kb, 2–3 kb. Within the gene boundary, sites were sectioned into 5′-UTR, 1^st^ intron, exons, other introns, 3′-UTR and 3 kb-downstream. Distal intergenic was defined as regions that are more than 3 kb from the gene boundary. Percentages are listed in the table. (**C**) Example of H3K27ac and PGR binding sites in the mouse genome visualised through UCSC Genome Browser. Tracks are located at chromosome 3 and have been normalised to the same scale. Genes and direction of transcription are indicated by black arrows. From top to bottom: H3K27ac (green track), PGR replicate 1 (orange track), PGR replicate 2 (orange track), input control (purple track). (**D**) Example of H3K27ac and PGR binding sites at the genomic region for *Mt2*. The red arrow indicates the TSS (arrow tail) and direction of transcription.

To identify the PGR-binding cistrome in granulosa cells we performed ChIP-seq on mouse granulosa cells isolated 6 h post-hCG. Between 17582 and 31958 peaks with sequence reads significantly above the baseline were identified in PGR ChIP-seq, with 15553 peaks common to both duplicates. These shared peaks represent the most reliable set of PGR binding sites and were assigned as the consensus PGR ChIP-seq data used for further analysis. When mapped to the mouse genome with distinct genomic annotations, these common peaks were assigned to 8656 genes, the majority (75.68%) being located within 3 kb of a transcription start site (TSS), consistent with a preferential interaction with gene promoters and potential impact on the expression of said gene (Fig. 2A). An accompanying H3K27ac ChIP-seq was performed in one of the granulosa chromatin samples to identify a genome-wide map of transcriptionally active promoter/enhancer regions. H3K27ac and PGR binding sites were mostly located in proximity to a TSS (Suppl. Data 4, left and middle panels). Global comparison of H3K27ac and PGR cistromes showed approximately three-quarters of PGR binding sites overlapping H3K27ac peaks, associated with 6385 genes (Fig. 2B) and suggesting that PGR mostly interacts with transcriptionally active regions. Indeed, 61.88% of the overlapping PGR and H3K27Ac peaks were situated in promoter regions, mostly within 1 kb of a TSS. An example of H3K27ac and PGR co-localisation is depicted in a section of chromosome 3, in which the majority of PGR binding sites overlapped with H3K27ac sites, especially in proximity to a genebody (Fig. 2C). This was also observed in PGR-regulated genes; for example in *Mt2*, a PGR-induced gene in granulosa cells as determined by microarray (Fig. 2D), prominent PGR peaks overlapping with H3K27ac peaks were detected surrounding the TSS of the gene, most notably in the proximal 5′ region, suggesting that this region is a PGR-responsive promoter important for *Mt2* transcription. Conversely, non-overlapping PGR or H3K27Ac peaks were relatively randomly distributed in relation to gene structures. In the case of PGR-unique peaks, nearly 40% of identified peaks fell within genebodies, mostly in intronic regions (Fig. 2B). Pathway and GO analyses of genes with H3K27ac and PGR binding sites also showed a high level of functional similarity, with pathways involving in cell cycle, transcription and translation regulation being enriched in both dataset (Suppl. Data 5, 6).

**Figure 3:**
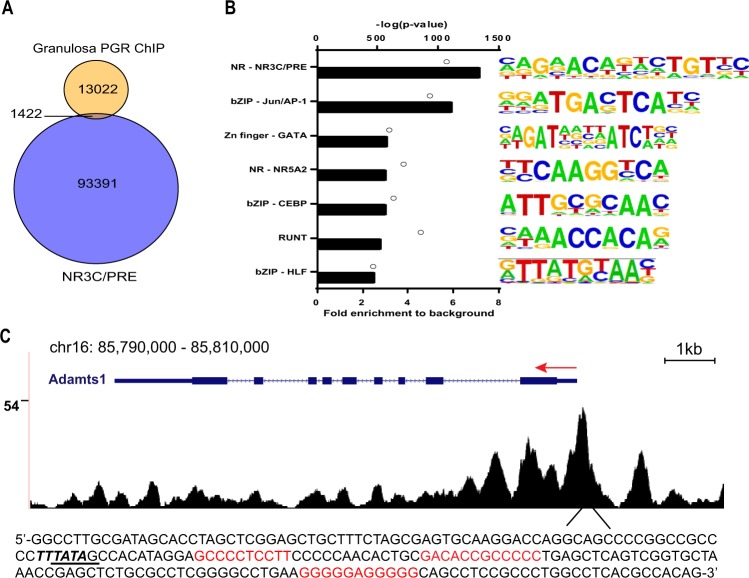
PGR binding is enriched at PRE/NR3C in the genome, but PGR also associates with other transcription factor binding elements. (**A**) PGR binding sites in granulosa cells in relation to global PRE/NR3C locations. PRE/NR3C sites within the entire mouse genome were identified using FIMO in MEME Suite to search for the consensus PRE/NR3C sequence. Analysis of enriched motifs represented in granulosa cell PGR ChIP data showed that 10.51% of peaks overlapped with consensus PRE/NR3C sites. (**B**) Top most common known motifs found to be enriched at PGR binding sites in granulosa cells. Bars indicate fold enrichment of motif to background. Circles indicate –log(p-value). Among motifs found by HOMER that belonged to the same transcription factor family, the most conservative sequences were selected and ranked by fold enrichment compared to background frequency. (**C**) PGR binding sites in the mouse genome visualised through UCSC Genome Browser. The track shown is located in chromosome 16 at the genomic region for *Adamts1*. The red arrow indicates the TSS (arrow tail) and direction of transcription. The sequence of the most prominent PGR peak is listed below, with the TATA box underlined and previously described G/C-site in red.

When comparing the PGR binding sites in granulosa cells against all 3-ketosteroid receptor motif (NR3C/PRE) loci in the mouse genome (94,813 loci), only 1422 (1.5%) were bound by PGR in granulosa cells. This represented approximately 10% of PGR binding peaks in granulosa cells (Fig. 3A), indicating that PGR-chromatin binding is not restricted to a recognised canonical NR3C/PRE. To determine other sequence motifs through which PGR commonly interacts in granulosa cells we performed motif finding in the PGR ChIP-seq peak dataset. The canonical NR3C/PRE sequence was the most enriched motif (7.19-fold) in the ChIP-seq data compared to the predicted random occurrence of this sequence motif (Fig. 3B, displayed as NR3C/PRE motif). Interestingly, a number of other TF binding motifs were also significantly enriched, albeit at lower level than NR3C/PRE. Among these were motifs for TF belonging to bZIP (AP-1 factors) (5.97-fold), GATA (3.1-fold), NR5A2 nuclear receptor (SF1/ LRH1) (3.07-fold), CEBP (3.07-fold), RUNT (2.82-fold) and HLF (2.55-fold) families (details in Suppl. Data 7). An example of a non-canonical PGR-binding region is shown for the *Adamts1* gene (Fig. 3C), a well characterised PGR-regulated gene in granulosa cells. Distinct PGR ChIP peaks were identified in the proximal promoter of *Adamts1*; however, the sequence at this site contains no identifiable NR3C/PRE motif. Rather, there were three G/C-rich boxes in the sequence previously determined to be Sp1/ Sp3-binding sites and were critical for the PGR-mediated induction of *Adamts1*^28^. Together these findings indicate that PGR selectively binds to a specific subset of NR3C/PRE in this target cell genome and also potentially acts in conjunction with other TF to regulate gene expression.

### Direct PGR binding is responsible for the regulation of PGR-dependent gene expression

**Figure 4:**
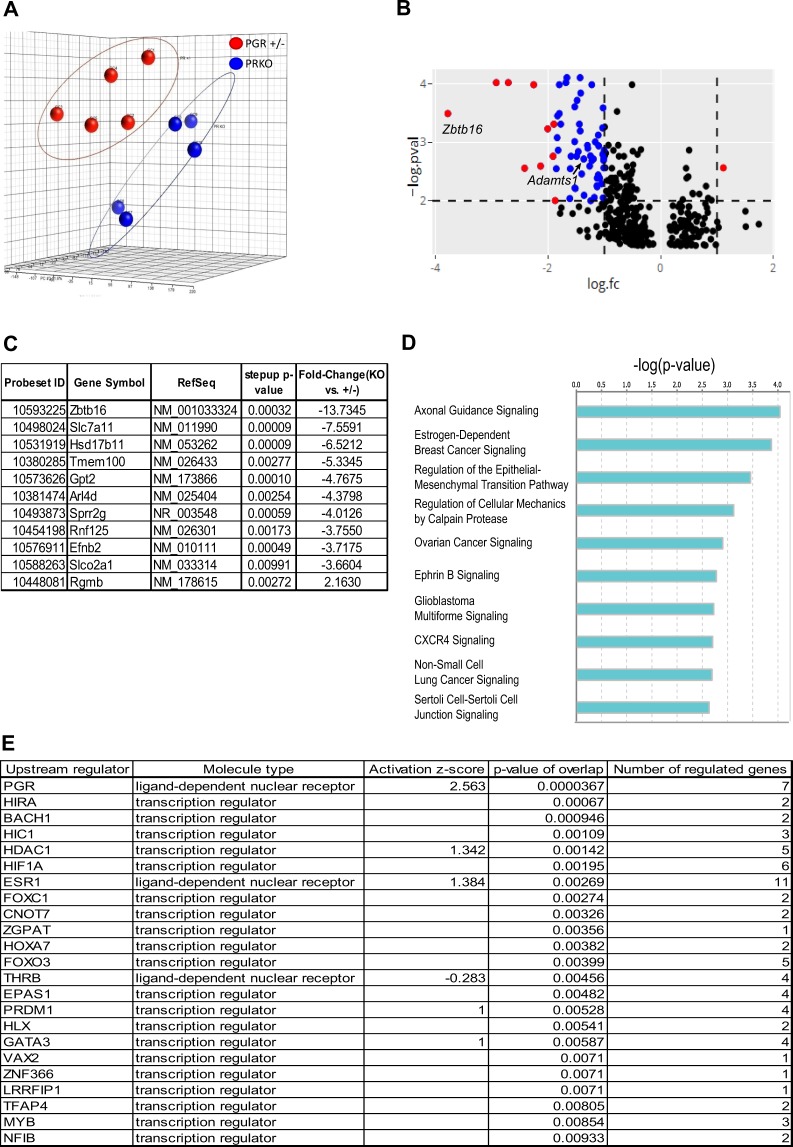
PGR-dependent differentially expressed genes in PRKO vs PGR +/−  peri-ovulatory granulosa cells. (**A**) Principal components analysis (PCA) plot of granulosa cell microarray samples. (**B**) Volcano plot of granulosa cell microarray data. A total of 367 genes with log fold change >1 or <−1 are plotted. The horizontal dashed line indicates p-value cut-off (p-value < 0.01, or –log(p-value) >2). The vertical dashed lines indicate fold change cut-off (logFC <−1 or >1). Genes that meet these criteria are indicated as blue symbols. Genes that have been selected for panel C are indicated as red symbols. (**C**) Examples of genes that are significantly differentially expressed in PRKO granulosa cells. DEG selected are genes that were differentially expressed (FC >2 or <−2) with a p-value cut-off of 0.01. These genes are annotated as red points in panel B. (**D**) Canonical pathway analysis of PGR-regulated DEG identified in microarray. 61 DEG that were determined from granulosa cell microarray were analysed for enriched pathways using the IPA software. Pathways with a -log(p-value) cut-off above 2 (or p-value < 0.01) were determined to be significantly enriched. (**E**) IPA upstream regulator analysis of genes identified in microarray, showing upstream regulators that were significantly enriched (p-value < 0.01).

PGR-dependent genes in granulosa cells were identified by microarray of PRKO vs PGR +/−  granulosa cell RNA collected 8 h post-hCG. In total more than 20000 genes were identified, which mostly corresponded to molecular binding activities and metabolic functions (Suppl. Data 8). Principle component analysis of the global gene expression profiles demonstrated a clear distinction between PGR +/−  vs PRKO peri-ovulatory granulosa cells (Fig. 4A). Applying criteria of FDR <0.01 and ≥2-fold change identified 61 genes with PGR-dependent expression (Suppl. Data 9). The vast majority of these (60 genes) were downregulated in PRKO granulosa cells, indicating that their expression is dependent on the presence of PGR (Fig. 4B,C). These included known PGR target genes *Zbtb16* and *Adamts1*. IPA also identified the canonical pathways most significantly represented in granulosa cell PGR-regulated genes, including pathways specific to reproductive tissues, such as estrogen-dependent breast cancer signalling and ovarian cancer signalling (Fig. 4D). Interestingly, CXCR4 signalling pathway was one of the most enriched pathways, which concurred with the fact that *Cxcr4* was identified as a PGR-dependent gene (Suppl. Data 9). Ingenuity pathway analysis (IPA) of upstream regulators confirmed that PGR was the most significant regulators of these genes (Fig. 4E).

**Figure 5:**
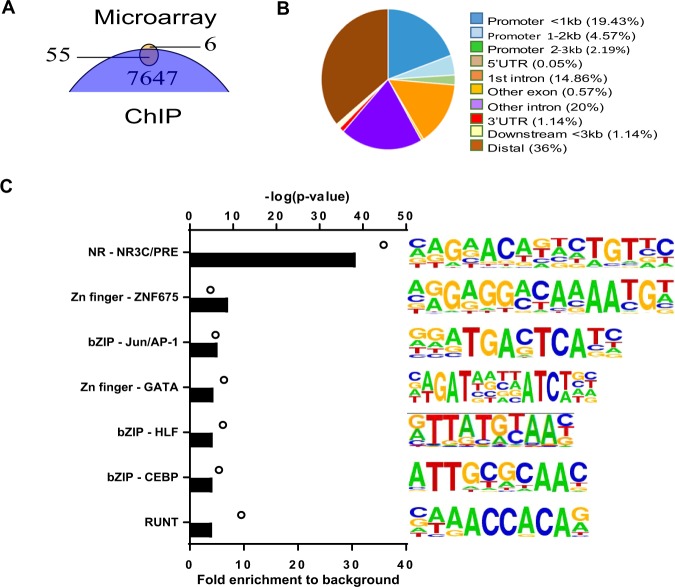
Characteristic of PGR-dependent DEG with PGR binding in peri-ovulatory granulosa cells. (**A**) Venn diagram showing DEG in microarray in relation to genes with PGR binding sites from ChIP-seq (**B**) Genome distribution of ChIP-seq peaks identified in PGR-dependent DEG. From (**A**), peaks that were found in annotated regions belonging to microarray-determined DEG were selected and analysed for genome distribution as defined in Fig. 2. (**C**) Top most common known motifs found to be enriched at PGR binding sites belonging to PGR-dependent DEG. Bars indicate fold enrichment to background. Circles indicate –log(pvalue). Among motifs found by HOMER that belonged to the same transcription factor family, the most conservative sequences were selected and ranked by fold enrichment compared to background frequency.

Comparison of DEG identified in PRKO granulosa cells with genes containing identified PGR peaks in ChIP-seq showed 55 of the 61 PGR-dependent genes in the microarray (82%) had at least one PGR-binding peak within or in proximity to the gene, accounting for 175 peaks in total (Fig. 5A). This suggests that direct PGR binding proximal to the gene location is a common mechanism responsible for PGR-dependent gene transcriptional regulation in granulosa cells. PGR-binding peaks associated with PGR-dependent genes were distributed throughout gene structures, with 27% being less than 3 kb from the TSS and more than two-thirds located within 3 kb of the gene (Fig. 5B). Motif analysis showed that among the PGR-regulated gene-associated peaks, NR3C/PRE was the most enriched, present in nearly 40% of all found peaks (30-fold enriched over predicted random occurrence) (Fig. 5C). Other motifs again included bZIP (AP-1), GATA, CEBP and RUNT motifs, similar to those identified in the whole ChIP-seq dataset (Fig. 3B).

### Tissue specificity of PGR-mediated transcription

**Figure 6:**
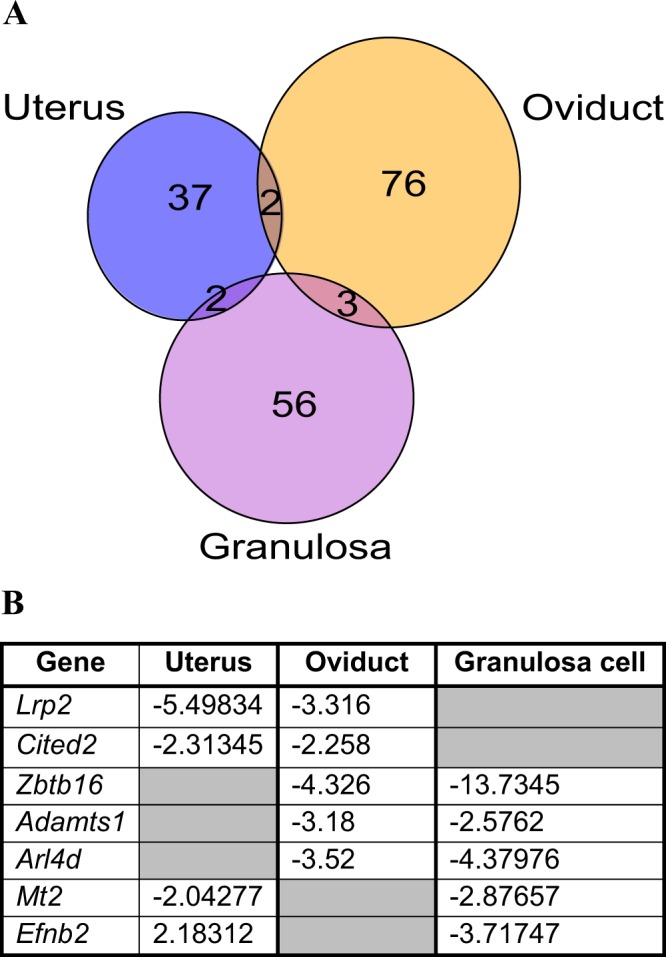
PGR regulates unique sets of genes that are tissue-specific. (**A**) Venn diagram of DEG identified in PRKO vs PGR + / ± uterus, oviduct and granulosa cells. DEG was compiled from independent microarrays performed on PRKO vs PGR + / ± uterus, oviduct and granulosa cells, selecting for genes that are differentially expressed (fold change >2 or <−2) with a p-value cut-off of 0.01. Graph was generated using VennDiagram package in R. (**B**) Overlapped genes that were identified in more than one tissue type, with accompanied PRKO to PGR + / ± fold change. A full list of DEG is available in Suppl. Data 5.

We next compared PGR dependent genes identified by microarray analysis of PRKO mouse uterus^22,23^ and oviduct^20^ with those in granulosa cells, using the same criteria for fold change and FDR which yielded 41 and 81 DEG, respectively (full gene list is available in Suppl. Data 9). There were few overlaps between PGR-dependent gene sets in the three tissues – only 7 genes were differentially expressed in more than one tissue and none was found to be shared in all three (Fig. 6A,B). Furthermore, one of these genes, *Efnb2*, was regulated in the inverse manner between granulosa cells and uterus, being significantly upregulated (2.2-fold) in PRKO uterus yet 3.7-fold downregulated in granulosa cells. GO analysis and IPA also showed a wide range of unique ontological terminologies and molecular pathways enriched in each PGR-regulated gene set (Suppl. Data 10, 11). This result confirms that PGR transcriptional regulation is highly diverse, specialised and tissue-dependent.

**Figure 7:**
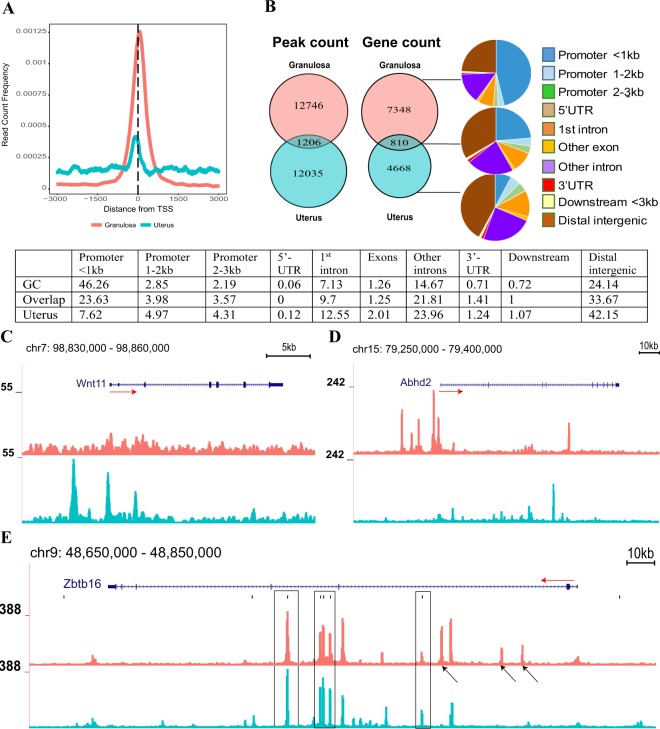
Comparative analyses of PGR binding sites in granulosa cells and uterus. (**A**) Read count frequency of PGR peaks in uterus (blue) and granulosa cells (pink) in relation to the TSS. (**B**) Venn diagram showing the peak count (left) and genes with peaks (right) of PGR binding in uterus and granulosa cells. Peaks were divided into granulosa-unique, overlapped and uterus-unique sections and analysed for genome distribution. The percentage of each region is listed in the below table. (**C**–**E**) PGR binding sites identified in PGR-regulated genes visualised in the mouse genome through UCSC Genome Browser for PGR peaks associated with uterus-regulated *Wnt11* (**C**), and with granulosa-regulated *Abhd2* (**D**) and *Zbtb16* (**E**). The top pink track represents peaks found in granulosa cells and the bottom blue track is for peaks in the uterus. Red arrows indicate TSS (arrow tail) and direction of transcription. In (**E**), black arrows indicate granulosa-specific peaks and black outline indicates peaks with PRE/NR3C motif.

While differences in PGR-regulated gene sets are not surprising considering different PGR physiological roles in different target tissues, we sought to investigate the mechanism of tissue-specific PGR actions by comparative analysis of granulosa cell and uterus ChIP-seq datasets. We re-analysed the uterus ChIP-seq raw data^31^ in parallel with our granulosa cell data using the latest mouse genome assembly and performed a comparative assessment of PGR binding sites in granulosa cell ChIP-seq against binding sites in the uterus after acute progesterone response. Both uterus and granulosa PGR sites were mostly centred at TSS (Fig. 7A); however, this occurrence was far more frequent in granulosa cells whereas in the uterus PGR was more commonly bound to chromatin sites distal to the TSS (Suppl. Data 4, middle and right panels). Comparison between uterus and granulosa cell peaks showed only 8.64% of peaks in granulosa cell and 9.11% of uterus peaks overlapped, totalling 810 annotated genes with PGR binding in common between the two tissues (Fig. 7B). The PGR-binding peaks specific to granulosa cells were more commonly found in proximal promoter regions (within 3 kb upstream from TSS – 51.3%), uterus specific PGR-binding sites were most enriched in distal regions (42.2%), while sites shared in both tissues were distributed relatively evenly in the genome, occupying with similar frequency the promoter, genebody or distal regions. For example, *Wnt11* whose expression is PGR-dependent in the mouse uterus (Suppl. Data 5), possessed significant PGR binding sites within 1 kb and 5 kb of the TSS as well as in the distal 5′-region almost 10 kb upstream of the gene boundary, which were not present in granulosa cells where *Wnt11* expression is not PGR-dependent (Fig. 7C). Contrastingly, *Abhd2*, a granulosa-specific PGR-regulated gene (Suppl. Data 5), showed multiple PGR binding sites located within the *Abhd2* promoter and 5′-distal region in granulosa cells that were not found in the uterus (Fig. 7D). PGR also bound to introns of many genes, such as in *Zbtb16*, a PGR-dependent gene in mouse granulosa cells (Fig. 4B, Suppl. Data 5) which has also been reported as PGR-induced in human and mouse endometrial stromal cells^33^. Despite *Zbtb16* being PGR-regulated in both tissues, the PGR interaction profile between the uterus and ovary was distinctive (Fig. 7E). While there were shared peaks between the two tissues, granulosa cells also exhibited a number of specific peaks and interestingly, only half of the discovered peaks had the consensus NR3C/PRE sequence. These intronic PGR-binding sites have been shown to be key regulatory elements in the uterine response to progesterone^33^. Genes associated with PGR binding in the two tissue types also belonged to different functional classifications (Suppl. Data 5).

**Figure 8:**
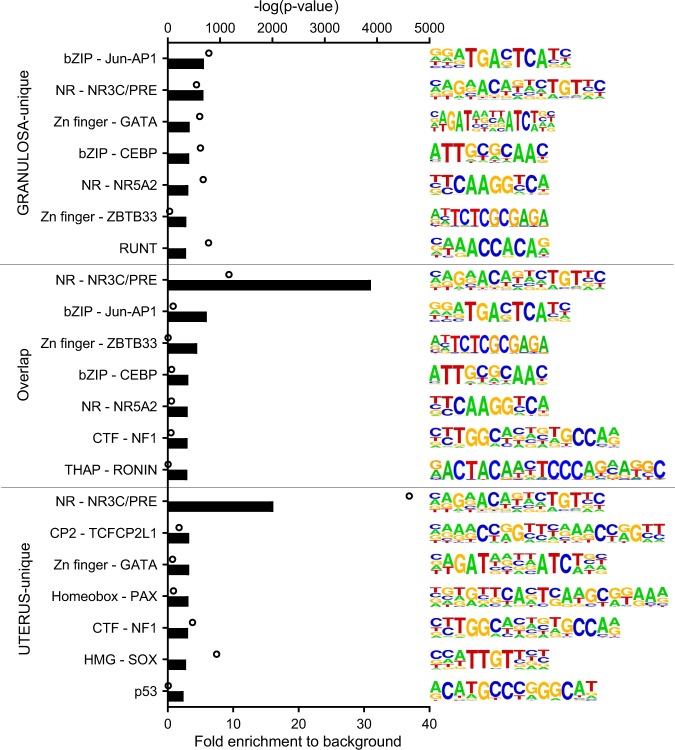
Tissue specificity of PGR binding motifs in granulosa cells and uterus. PGR binding sites were divided by specificity to either granulosa cells, uterus or present in both (as in Fig. 7B) for motif analysis. Bars indicate fold enrichment to background. Circles indicate –log(pvalue). Among motifs found by HOMER that belong to the same TF family, the most conservative sequences are selected and ranked by fold enrichment compared to background frequency.

HOMER motif analysis comparing sequences that were enriched at PGR binding sites in each tissue identified the consensus NR3C/PRE to be most highly enriched (31-fold over predicted random occurrence) in granulosa- and uterus-shared ChIP-seq peaks. Interestingly, in peaks specific to uterus, consensus NR3C/PRE was more highly enriched compared to peaks specific to granulosa cells (16.13-fold vs 5.47-fold, respectively) (Fig. 8). Additional differences were evident in non-NR3C/PRE motifs enriched in PGR ChIP-seq from either tissue type. PGR peaks in granulosa cells included strongly enriched motifs for bZIP (AP-1), Zn finger TF, CEBP, NR5A2 and RUNT families. Conversely, in the uterus, relatively modest enriched motifs belonged to CP2, Homeobox and SOX families. This suggests that PGR activity is much more dependent on direct PGR binding to NR3C/PRE in the uterus and PGR function may rely on tissue-specific interaction with different TF in different tissue context.

## Discussion

It is known that PGR plays diverse roles in reproductive tissues, enabling P4-PGR signalling to coordinate various physiological processes in female reproduction. However, few attempts have been made to describe the molecular mechanisms that allow PGR to achieve pleiotropic actions in the reproductive tract. In this study we have established the first genome-wide characterisation of PGR action in granulosa cells and contrasted this with the uterus to show that PGR is responsible for the regulation of tissue-specific genes through selective interaction with distinct NR3C/PRE in the genome of each cell type and with novel accessory TF.

One of our major findings is the discovery of possible cell-specific mechanisms through which PGR interacts with target genomes. In all target tissues PGR like all NR3C receptors including glucocorticoid receptor (GR) and androgen receptor (AR), binds to the core motif – 5′-GnACAnnnTGTnC-3′ (hormone response element, HRE). In granulosa cells, this consensus NR3C/PRE motif dominated among the genome-wide PGR-bound sequences, implying that the main mode of PGR function is through directly binding chromatin through the canonical HRE. However, in PGR binding intervals unique to the uterus, or shared between uterus and granulosa cells, the NR3C/PRE binding motif and NR3C/PRE half sites were highly represented, while in comparison these motifs were far less common in granulosa-specific intervals. Considering that >98% of NR3C/PRE consensus motifs in the mouse genome were not utilised in either tissue and most NR3C/PRE bound by PGR were unique to either granulosa cells or uterus, it is clear that PGR chromatin occupancy is highly selective to specific NR3C/PRE in the genome of different tissues and that PGR frequently interacts with sites in the mouse granulosa cell genome that do not possess the NR3C/PRE consensus sequence.

The range of non-NR3C/PRE motifs enriched in the PGR-bound intervals included those for AP-1, GATA, NR5A2 and RUNT families. Our findings indicate that unique interactions of PGR with AP-1, NR5A2, RUNT and GATA may each contribute to the granulosa specific PGR action in ovulation. These partner transcription factors potentially regulate granulosa-specific chromatin remodelling to establish accessibility to specific NR3C/PRE (i.e. pioneer factors), or through direct protein-protein interactions with PGR; suggesting that in granulosa cells, PGR not only utilises direct NR3C/PRE-binding to interact with target chromatin but also that tethering of PGR to target chromatin domains through alternative accessory transcription factors is responsible for the functional diversity in granulosa cells. As motif analysis only acts as a screening method for potential interacting partners, these hypotheses will need to be confirmed with assays of protein-protein interaction, such as immunoprecipitation or proximity ligation assay. Supporting this hypothesis we now demonstrated the previously hypothesised binding of PGR to specific G/C-rich sites in the *Adamts1* gene promoter, which contains no PRE motif but has been shown through promoter-reporter analysis to be required for PGR-dependent induction of *Adamts1* transcription^28^. This model of NR-comodulator cooperation has been previously described for GR and AR. Specifically, GR transcriptional regulation in endometrial cells is also shown to rely on association with pioneer factors, including FOXA1 along with the canonical HRE^34,35^ and likewise, the switch in AR action in normal versus malignant prostate cancer is related to context-specific interactions with FOXA1 or AP-1 co-regulators^36,37^. Recent studies of PGR in progesterone-primed uterus have indicated GATA2 as a candidate partner of PGR action in uterine tissue^23^. Consistent with our findings, a similar study comparing PGR cistromes between T47D breast cancer cell line versus primary leiomyoma found less than 15% overlap in PGR-binding sites, and frequent PGR binding within 5 kb of TSS in T47D cells was likewise attributed to context-specific accessory factors who’s motif sequences were enriched in the PGR binding intervals^38^. Taken with these previous reports of NR tethering to non-canonical motifs through accessory factors, our findings support a mechanism whereby granulosa-specific PGR action is mediated by interaction of PGR with AP-1, NR5A2 and RUNT TFs. Interestingly, members of each of these families are strongly induced in peri-ovulatory granulosa cells and are known to participate in the ovulation pathway^39–42^. Also noteworthy are the recent findings that the RUNX transcription regulating cofactor CBFb is required for ovulatory gene expression^43^ and that PGR participates in peri-ovulatory induction of with AP-1 family members Fos, JunB and JunD^44^.

Our data show that PGR exerts diverse physiological roles in different target organs through the regulation of widely different sets of genes. PGR also possesses a remarkable tissue-specific inclination for binding to specific gene regions depending on the tissue type. In granulosa cells PGR preferentially bound very proximal gene promoter regions. Over 50% of granulosa specific PGR-binding intervals were within 3 kb upstream of a TSS, while in the uterus binding to intergenic chromatin was more common. The strong association between PGR and H3K27ac binding events indicates that PGR is involved with transcriptionally active regions. That there is a comparatively large enrichment of promoter binding in granulosa cells suggests possible alternative mechanisms through which PGR regulates gene expression in each target tissue. PGR is only transiently present in granulosa cells, which may bias chromatin binding towards the most available open chromatin regions in key granulosa cell target genes, i.e. an accessible chromatin where a pioneer TF has already bound. In comparison, in the uterus where PGR is constitutively present, P4-activated PGR more commonly binds to distal intergenic regions^31^. Thus in the uterus, PGR may contribute to forming chromatin topological structure and segmenting the genome into a permissive 3-dimensional functional structure (chromatin looping), primed for a specific response to P4 ligand which is provided by ovarian granulosa cells at ovulation^45^.

Our study is the first to define the genome-wide PGR cistrome in granulosa cells responding to the ovulatory signal, when PGR induction and its response to P4 are essential for ovulation and hence fertility. The application of genome-wide microarray in PRKO and PGR ChIP-seq in granulosa cells along with comparative analysis to P4-responsive genes in the uterus and oviduct allows us to fully dissect the unique intricate details of transcriptional regulation specific to ovulation. As the complexities of transcriptional regulation are revealed, it is apparent that the participation of non-promoter sequences such as enhancers and distal intergenic regions also play a vital role in modulating transcription of distal genes. In addition modulating interactions between transcriptional regulators and protein or nucleotide (i.e. ncRNA) co-factors, is critical. Thus, it is important to investigate the transcriptional complex as a whole in order to gain a full comprehension of how gene expression is regulated in each cell context. The present insights into PGR action in granulosa cells deepen our understanding of tissue-specific mechanisms of PGR in ovulation, dysregulation of which is likely to contribute to the aetiology of anovulatory infertility. Conversely, understanding this mechanism can also lead to novel targets for the development of new specific contraceptives that block ovulation. Alternatively, by adding to the landscape of PGR responsiveness in reproductive tissues, our findings can lead to implications on new and efficient cancer therapeutics targeting specific reproductive organs with mitigated side effects on other PGR-dependent tissues.

## Methods

### Reagents and antibodies

Unless otherwise stated, reagents were purchased from Sigma-Aldrich (St. Louis, MO, USA). Antibodies used for ChIP were for PGR (sc-7208, Santa Cruz Biotechnology, Texas, USA) and H3K27ac (#39133, Active Motif, Carlsbad, USA). Primary antibodies used for Western blot were for PGR (MA5-12658, ThermoFisher, Scoresby, Australia) and H3 (#9715, Cell Signaling, Danvers, USA). Fluorescent secondary antibodies for Western blot were goat anti-rabbit (926–32211, Li-Cor Biotechnology, Lincoln, USA) and donkey anti-mouse (925–32212, Li-Cor).

### Animals and hormone induction of ovulation

21 day old CBA × C57BL/6 F1 mice were obtained from The University of Adelaide, Laboratory Animal Services. All mice were maintained in 12 h/ 12 h light/ dark conditions and given water and rodent chow *ad libitum*. PGR +/−  and PRKO mice generation and genotyping were as previously described^20^. Female PGR +/−  mice display normal ovulation and fertility. All experiments were approved by The University of Adelaide Animal Ethics Committee and were conducted in accordance with the Australian Code of Practice for the Care and Use of Animals for Scientific Purposes. For the time-course experiment, mice were untreated or were hormonally stimulated by intraperitoneal injection with 5 IU equine chorionic gonadotropin (eCG) following by 5IU hCG at 46 hour-post eCG. Mice were culled at the following time points: unstimulated (no eCG and hCG), 0 h (no hCG), 4 h, 6 h, 8 h, 10 h, 12 h post-hCG. Ovaries were dissected and granulosa cells were collected by repeated puncturing of the ovaries. Depending on the time point, granulosa cells from 1–3 ovaries were pooled together for RNA extraction and granulosa cells from 3 ovaries were pooled for Western blot. A total of three independent experimental replicates were conducted for mRNA and protein quantification.

### PGR mRNA quantification

RNA was extracted from granulosa cells using RNeasy Mini kit (Qiagen, Chadstone, VIC, Australia) as per manufacturer’s protocol. cDNA was synthesised from 500 ng extracted RNA using SuperScriptIII Reverse Transcriptase kit (Thermo Fisher). cDNA was used for qPCR with Taqman methodology (assays: *Pgr* – Mm00435628_m1, *Rpl19* – Mm02601633_g1, Thermo Fisher). Each sample was run in technical triplicates. *Pgr* expression in each biological replicate was normalised to *Rpl19* and fold change was presented as relative to the mean of unstimulated samples using the ddCT method. Results were presented as mean ± SEM. One-way analysis of variance (ANOVA) was used as indicated in the figure legends and statistical significance was considered as p-value < 0.05.

### PGR protein quantification

Isolated granulosa cells were lysed in LDS buffer (Thermo Fisher) containing 1 µl β-mercaptoethanol and heated to 65 °C for 10 minutes. Equal volume of samples were loaded and protein separation was achieved by gel electrophoresis at 165 V for 45 minutes. Protein was transferred onto nitrocellulose membrane and blocked in Odyssey Blocking buffer (Li-Cor). Primary and corresponding secondary antibodies were diluted 1:1000 in blocking buffer and incubated with membranes at 4 °C for 1 hour. Membranes were imaged using Odyssey Imager for fluorescent antibody detection.

### Microarray data analysis

Gene lists for oviduct and uterus microarray on PRKO vs PGR + / ± mice were from previously published datasets^20,22,23^. Comparison of differentially expressed genes in PRKO vs PGR + / ± granulosa cells was performed by microarray of RNA isolated from granulosa cells collected after 8 h hCG treatment, which is within 2 h of peak PGR expression in the granulosa cells. Five replicate pools of granulosa cell RNA from 3 animals per replicate were generated with RNA integrity thresholds of 8.1–9.6 verified using an Agilent Bioanalyzer. Microarray was performed and analysed as previously described^20^. Briefly, 100 ng of total RNA was hybridised to Affymetrix GeneChip Mouse Gene 1.0 ST Arrays (Affymetrix) and scanned on Affymetrix GeneChip scanner 3000 7 G Plus. Background correction, probe affinity adjustment, quantile normalisation and data analysis were performed using Partek Genomics Suite software (Partek). Full datasets have been deposited in the National Center for Biotechnology Information (NCBI) Gene Expression Omnibus (GEO: GSE92438). Upstream regulator analysis was performed using IPA software (Qiagen) and regulators belonging to category ‘ligand-dependent nuclear receptor’ and ‘transcription regulator’ were selected. FDR <0.01 and fold change >2 criteria were applied to obtain significantly differentially expressed genes (DEG) for subsequent comparisons. Gene Ontology (GO) analysis was performed using R packages and canonical pathway analysis was performed using IPA software.

### Chromatin immunoprecipitation-sequencing (ChIP-seq)

Granulosa cells were collected by puncturing ovarian follicles 6 h after hCG stimulation at peak PGR protein abundance. Two biological replicates were obtained from 10 mice, each with at least 1 × 10^7^ cells. ChIP-seq for PGR (2 replicates) and H3K27ac (1 replicate) was performed by Active Motif. Briefly, cells were fixed in 1% formaldehyde for 15 minutes and quenched with 0.125 M glycine. Chromatin was isolated by the addition of lysis buffer, followed by disruption with a Dounce homogenizer. Lysates were sonicated and the DNA sheared to an average length of 300–500 bp. Lysate was precleared with protein A agarose bead (Invitrogen, Waltham, USA) and PGR ChIP was performed using 4 µg antibody with 30 µg (for PGR) or 20 µg (for H3K27ac) chromatin. Protein-chromatin complexes were washed, eluted from beads and subjected to RNase and proteinase K treatment. Reverse crosslinking was through overnight incubation at 65 °C. DNA was purified by phenol-chloroform extraction and ethanol precipitation. Isolated chromatin was confirmed using qPCR on specific genomic regions with expected PGR and H3K27ac interaction in triplicate using SYBR Green Supermix (Bio-Rad, Hercules, USA). Illumina sequencing libraries were prepared from the ChIP and input DNA by the standard consecutive enzymatic steps of end-polishing, dA-addition, and adaptor ligation. After a final PCR amplification step, the resulting DNA libraries were quantified and sequenced on Illumina’s NextSeq 500 (75 nt reads, single end).

### ChIP data analysis

PGR and H3K27ac ChIP-seq data for granulosa cell was generated as above (GEO: GSE115820) and data for uterus PGR ChIP-seq was obtained from the publicly available database (GEO: GSE34927)^31^. For all datasets, 75-base sequences were aligned against the current mouse genome assembly (mm10) using Bowtie2 algorithm. Peak calling from read count followed the algorithm for Model-based Analysis for ChIP-Seq (MACS2) with a p-value cut-off = 10^−10^ and a mouse genome size of 1.87 × 10^9^. For granulosa cell PGR ChIP, initial analysis of both replicates showed a high level of overlap, with the majority of peaks called in PGR-2 identifiable in PGR-1. Thus, the overlapped peaks were used as the consensus data in all subsequent comparisons. This was determined using ChIPseeker R/Bioconductor package^46^ and overlapped peaks were chosen as peaks with narrower width for a more conservative dataset. For genome localisation, gene boundaries were set as ± 3 kb of the TSS/gene end, with TSS – 3 kb assigned as promoter and gene + 3 kb assigned as downstream. Regions that did not fall into this range were considered to be distal intergenic. Absolute distance of peaks to TSS and pathway enrichment analysis was through GREAT analysis for mouse mm10 genome assembly, whole genome as background and at default settings^47^. Gene Ontology (GO) analysis was performed using R packages like for microarray gene analysis. Motif analysis for known and *de novo* sequence motifs was performed using HOMER motif finding algorithm^48^, with random 200 bp-long sequences from the mouse genome used to estimate motif frequency in random sequence (motif enrichment over background). Global NR3C/PRE sites in the mouse genome were identified using the FIMO tool from MEME Suite^49^ with the consensus full-length NR3C/PRE sequence from HOMER Motif Database (Suppl. Data 1). The complete R script used for analysis is included in Suppl. Data 2.

## Supplementary information

Supplementary Data

Supplementary Data 2

## Data Availability

The datasets generated and/or analysed during the current study are available in the NCBI Gene Expression Omnibus repository: GSE115820 (granulosa cell ChIP-seq), GSE34927 (uterus ChIP-seq), GSE92438 (granulosa cell microarray), GSE51499 (oviduct microarray), GSE39920 (uterus microarray).
