# Supplementary material for: Tissue-specific progesterone receptor-chromatin binding and the regulation of progesterone-dependent gene expression
Source: Sci Rep. 2019 Aug 19;9:11966. doi: 10.1038/s41598-019-48333-8 (PMC6700090; doi:10.1038/s41598-019-48333-8)
Supplement: Supplementary file 1 — Supplementary Data [file 41598_2019_48333_MOESM1_ESM.pdf]

Tissue-specific progesterone receptor-chromatin binding and the regulation of progesterone-dependent gene expression

Dinh DT [1], Breen J [1,2], Akison LK [3], DeMayo FJ [4], Brown HM [1,5,6], Robker RL [1] & Russell DL* [1]

[1] Robinson Research Institute, Adelaide Medical School, University of Adelaide, Australia

[2] University of Adelaide Bioinformatics Hub, University of Adelaide, Australia

[3] Child Health Research Centre, Centre for Children's Health Research, The University of Queensland, South Brisbane, Qld 4101, Australia.

[4] Pregnancy and Female Reproduction Group, National Institute of Environmental Health Sciences, Research Triangle Park, NC 27709.

[5] South Australian Health and Medical Research Institute, Adelaide, South Australia, Australia

[6] Australian Research Council (ARC) Centre for Nanoscale Biophotonics, University of Adelaide, Australia

Supplementary data 1: Input sequence for PRE motif finder with FIMO – MEME Suite. The letter probability matrix was obtained from HOMER motif database (http://homer.ucsd.edu/homer/custom.motifs - ID: PGR(NR)/EndoStromal-PGR-ChIP-Seq(GSE69539)/Homer) and used as input for the web-based FIMO program from MEME Suite for identification of global PRE sites in whole mouse genome.

MEME version 4
ALPHABET= ACGT
strands: + -
Background letter frequencies (from unknown source):
A 0.250 C 0.250 G 0.250 T 0.250
MOTIF 1 NRGWACANWNTGTHC
letter-probability matrix: alength= 4 w= 15 nsites= 1 E= 0e+0
0.413000 0.186000 0.190000 0.211000
0.715000 0.001000 0.244000 0.040000
0.112000 0.001000 0.886000 0.001000
0.639000 0.087000 0.117000 0.157000
0.997000 0.001000 0.001000 0.001000
0.001000 0.997000 0.001000 0.001000
0.919000 0.001000 0.001000 0.079000
0.200799 0.171828 0.225774 0.401598
0.383000 0.113000 0.109000 0.395000
0.339339 0.303303 0.146146 0.211211
0.054000 0.001000 0.001000 0.944000
0.001000 0.001000 0.997000 0.001000
0.001000 0.001000 0.001000 0.997000
0.179000 0.146000 0.066000 0.609000
0.025000 0.865000 0.001000 0.109000

Supplementary data 2: R script used for bioinformatics analysis of ChIP-seq data. The script is presented as an Rmarkdown file.

See Supplementary data 2

Supplementary data 3: Full-length Western blot of PGR in granulosa cells during ovulation (unstim or eCG + hCG 0-12 h post-hCG), from Fig 1B. Western blot was performed in biological triplicates, with 3-4 mice per time point in each replicate. White line indicates cut line for multiple immunoblotting on the same membrane. One representative example of three highly similar results is shown.

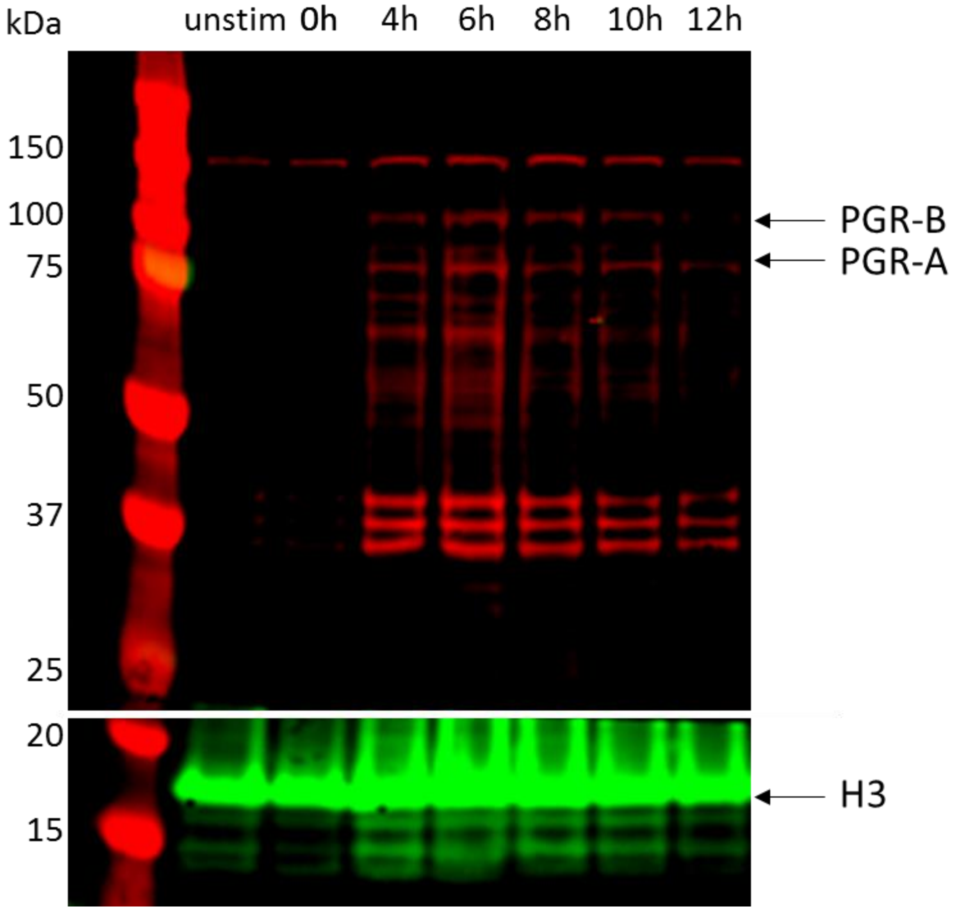

Supplementary data 4: (A) Binding sites for H3K27ac (green), PGR in granulosa cells (orange) and PGR in uterus (blue) in relation to absolute distance to TSS. (B) Heatmap indicating the general distribution of binding sites of H3K27ac (green), PGR in granulosa cells (orange) and in the uterus (blue) within 3 kb to the TSS.

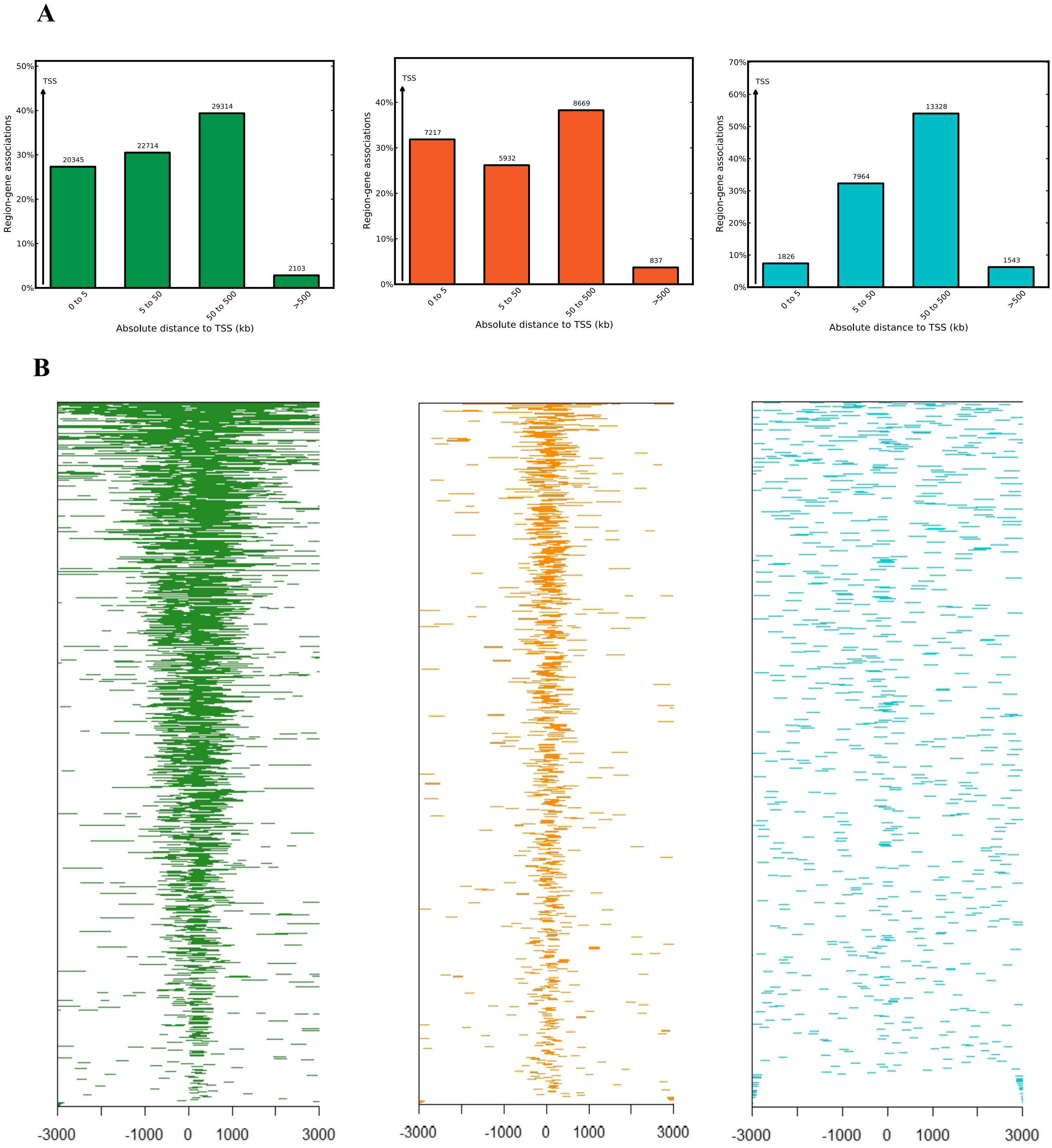
A
B
TSS
Region-gene associations
Absolute distance to TSS (kb)
0 to 5
5 to 50
50 to 500
>500
0%
10%
20%
30%
40%
50%
60%
70%
20345
22714
29314
2103
7217
5932
8669
837
1826
7964
13328
1543
-3000
-1000
0
1000
3000

Supplementary data 5: Top ten most enriched pathways of binding sites for H3K27ac (green), PGR in granulosa cells (orange) and in the uterus (blue). Bars indicate fold enrichment of pathways. Circles indicate –log(FDR) value. Analysis was performed using GREAT analysis for enrichment over the mm10 genome background.

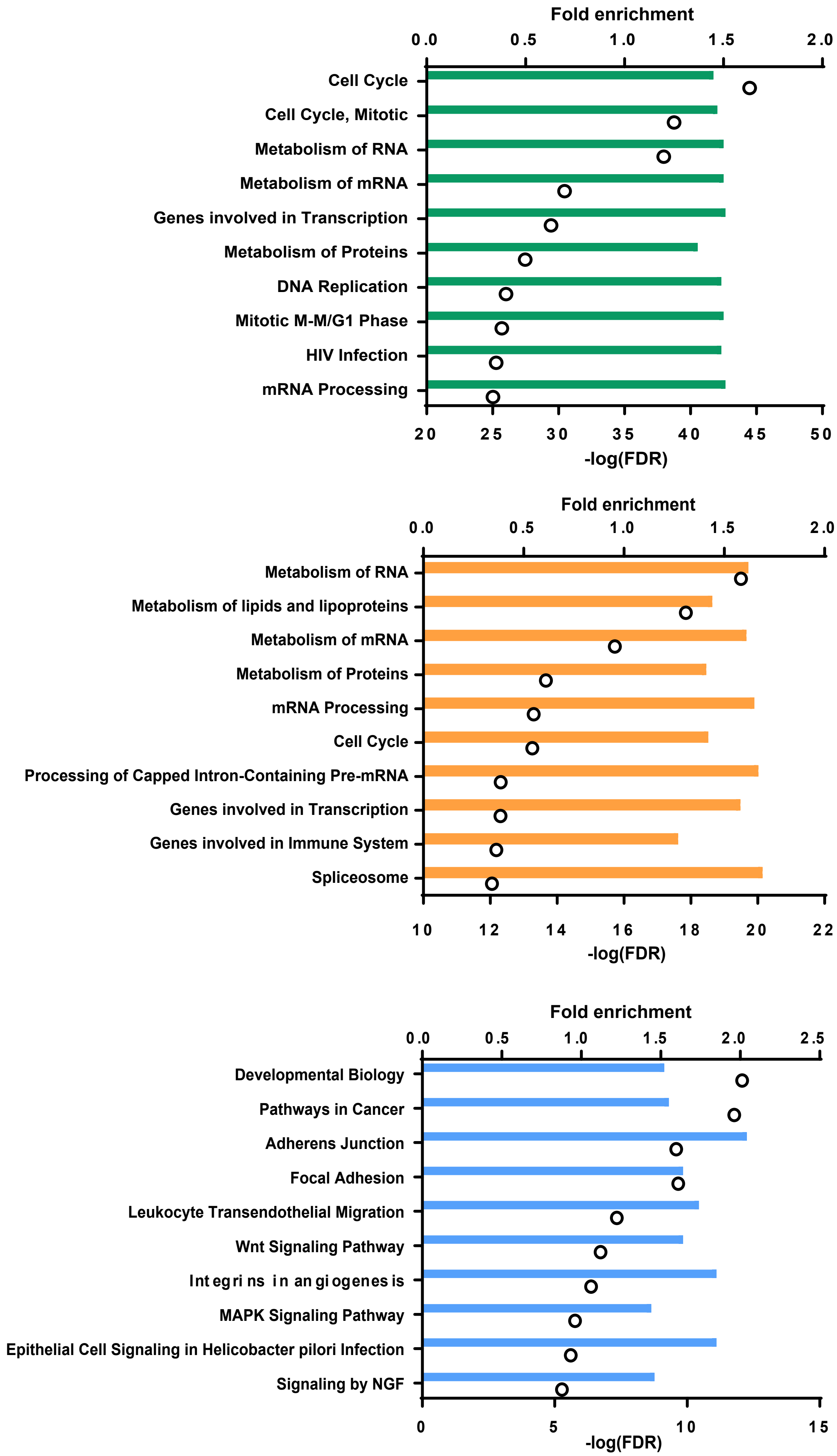

Fold enrichment
0.0
0.5
1.0
1.5
2.0
Cell Cycle
Cell Cycle, Mitotic
Metabolism of RNA
Metabolism of mRNA
Genes involved in Transcription
Metabolism of Proteins
DNA Replication
Mitotic M-M/G1 Phase
HIV Infection
mRNA Processing
20
25
30
35
40
45
50
-log(FDR)
Fold enrichment
0.0
0.5
1.0
1.5
2.0
Metabolism of RNA
Metabolism of lipids and lipoproteins
Metabolism of mRNA
Metabolism of Proteins
mRNA Processing
Cell Cycle
Processing of Capped Intron-Containing Pre-mRNA
Genes involved in Transcription
Genes involved in Immune System
Spliceosome
10
12
14
16
18
20
22
-log(FDR)
Fold enrichment
0.0
0.5
1.0
1.5
2.0
2.5
Developmental Biology
Pathways in Cancer
Adherens Junction
Focal Adhesion
Leukocyte Transendothelial Migration
Wnt Signaling Pathway
Integrins in angiogenesis
MAPK Signaling Pathway
Epithelial Cell Signaling in Helicobacter pilori Infection
Signaling by NGF
0
5
10
15
-log(FDR)

Supplementary data 6: GO analysis of genes with H3K27ac, PGR binding sites in granulosa cells and in the uterus. Genes were annotated for ChIP-seq binding sites and subjected to GO analysis using the *limma* Bioconductor package in R with mm10 terminology and a FDR = 0.05 cut-off. The ten most significantly enriched GO terms from each GO category were selected as representation.

	Dataset	GO ID	Term	Total genes in pathway	Gene matching term in dataset	p-value
Biological Process	Granulosa cell - H3K27ac	nitrogen compound metabolic process	GO:0006807	8771	5750	0
		metabolic process	GO:0008152	10337	6582	0
		cellular process	GO:0009987	15608	8601	0
		macromolecule metabolic process	GO:0043170	8357	5367	0
		cellular metabolic process	GO:0044237	9199	6165	0
		primary metabolic process	GO:0044238	9296	6026	0
		cellular macromolecule metabolic process	GO:0044260	6974	4716	0
		organic substance metabolic process	GO:0071704	9839	6253	0
		cellular aromatic compound metabolic process	GO:0006725	4913	3456	0
		cellular nitrogen compound metabolic process	GO:0034641	5435	3837	0
	Granulosa cell - PGR	cellular metabolic process	GO:0044237	9199	4106	0
		metabolic process	GO:0008152	10337	4384	6.65E-302
		primary metabolic process	GO:0044238	9296	4021	2.68E-284
		organic substance metabolic process	GO:0071704	9839	4186	6.28E-282
		nitrogen compound metabolic process	GO:0006807	8771	3820	2.33E-269
		cellular process	GO:0009987	15608	5729	3.66E-235
		cellular macromolecule metabolic process	GO:0044260	6974	3140	2.78E-231
		macromolecule metabolic process	GO:0043170	8357	3572	2.81E-221
		cellular nitrogen compound metabolic process	GO:0034641	5435	2565	1.05E-212
		organic cyclic compound metabolic process	GO:1901360	5097	2375	6.56E-183
	Uterus - PGR	localization	GO:0051179	5794	1671	8.68E-106
		cellular process	GO:0009987	15608	3537	6.60E-105
		cellular metabolic process	GO:0044237	9199	2330	1.59E-92
		metabolic process	GO:0008152	10337	2537	1.46E-88
		developmental process	GO:0032502	6097	1676	2.74E-86
		anatomical structure development	GO:0048856	5712	1591	1.97E-85
		primary metabolic process	GO:0044238	9296	2320	3.55E-84
		organic substance metabolic process	GO:0071704	9839	2422	3.75E-83
		system development	GO:0048731	4638	1348	9.23E-83
		multicellular organism development	GO:0007275	5227	1474	1.19E-81
Molecular Function	Granulosa cell - H3K27ac	binding	GO:0005488	13121	7816	0
		protein binding	GO:0005515	8965	5596	0
		heterocyclic compound binding	GO:1901363	4989	3481	0
		ion binding	GO:0043167	5386	3576	3.72E-264
		enzyme binding	GO:0019899	2282	1726	1.44E-205
		nucleic acid binding	GO:0003676	3093	2172	1.16E-191
		nucleotide binding	GO:0000166	2003	1504	7.91E-172
		nucleoside phosphate binding	GO:1901265	2003	1504	7.91E-172
		transferase activity	GO:0016740	2256	1644	2.08E-166
		small molecule binding	GO:0036094	2381	1694	1.92E-154
	Granulosa cell - PGR	binding	GO:0005488	13121	5170	1.76E-287
		protein binding	GO:0005515	8965	3732	9.34E-208
		heterocyclic compound binding	GO:1901363	4989	2317	7.71E-175
		organic cyclic compound binding	GO:0097159	5086	2346	1.07E-172
		catalytic activity	GO:0003824	5747	2563	1.68E-167

		ion binding	GO:0043167	5386	2379	2.16E-145
		nucleic acid binding	GO:0003676	3093	1451	5.35E-104
		enzyme binding	GO:0019899	2282	1145	2.69E-103
		metal ion binding	GO:0046872	3445	1520	6.86E-84
		cation binding	GO:0043169	3542	1551	7.68E-83
	Uterus - PGR	binding	GO:0005488	13121	3182	1.64E-130
		protein binding	GO:0005515	8965	2355	9.67E-115
		ion binding	GO:0043167	5386	1505	1.41E-80
		catalytic activity	GO:0003824	5747	1505	7.07E-59
		cation binding	GO:0043169	3542	992	4.36E-49
		enzyme binding	GO:0019899	2282	703	5.95E-49
		metal ion binding	GO:0046872	3445	964	2.51E-47
		anion binding	GO:0043168	2640	777	5.78E-46
		organic cyclic compound binding	GO:0097159	5086	1258	9.54E-34
		heterocyclic compound binding	GO:1901363	4989	1228	9.09E-32
Cellular Component	Granulosa cell - H3K27ac	intracellular	GO:0005622	13641	9072	0
		cell	GO:0005623	16002	9608	0
		cytoplasm	GO:0005737	10813	7286	0
		intracellular part	GO:0044424	13571	9034	0
		cytoplasmic part	GO:0044444	8210	5702	0
		cell part	GO:0044464	15998	9607	0
		organelle	GO:0043226	12153	8240	0
		membrane-bounded organelle	GO:0043227	10873	7550	0
		intracellular organelle	GO:0043229	11843	8126	0
		intracellular membrane-bounded organelle	GO:0043231	10153	7215	0
	Granulosa cell - PGR	intracellular	GO:0005622	13641	5939	0
		cell	GO:0005623	16002	6315	0
		cytoplasm	GO:0005737	10813	4800	0
		intracellular part	GO:0044424	13571	5915	0
		cytoplasmic part	GO:0044444	8210	3765	0
		cell part	GO:0044464	15998	6314	0
		organelle	GO:0043226	12153	5395	0
		membrane-bounded organelle	GO:0043227	10873	4956	0
		intracellular organelle	GO:0043229	11843	5324	0
		intracellular membrane-bounded organelle	GO:0043231	10153	4733	0
	Uterus - PGR	cell	GO:0005623	16002	3821	1.16E-198
		cell part	GO:0044464	15998	3820	1.82E-198
		intracellular	GO:0005622	13641	3402	3.79E-182
		intracellular part	GO:0044424	13571	3385	5.87E-180
		cytoplasm	GO:0005737	10813	2813	5.99E-150
		organelle	GO:0043226	12153	3046	2.52E-144
		intracellular organelle	GO:0043229	11843	2984	2.68E-142
		membrane-bounded organelle	GO:0043227	10873	2699	1.28E-106
		cytoplasmic part	GO:0044444	8210	2165	3.15E-102
		intracellular membrane-bounded organelle	GO:0043231	10153	2492	8.43E-86

Supplementary data 7: HOMER output for known motif enrichment analysis of PGR binding sites in granulosa cells. Motifs are ranked by fold change to background and only motifs with a fold change ≥ 2 are included.

Supplementary data 7

HOMER output

Total Target Sequences = 12553, Total Background Sequences = 36232

Motif	Name	log P-pvalue	# Target Sequences with Motif	% of Targets Sequences with Motif	# BG Sequences with Motif	% of BG Sequences with Motif	FC to BG
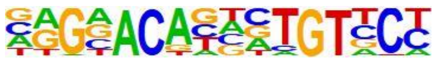	GRE(NR),IR3/A549-GR-ChIP-Seq(GSE32465)/Homer	-1067	930	7.41%	372.3	1.03%	7.19
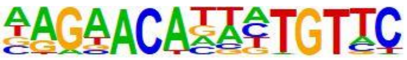	PGR(NR)/EndoStromal-PGR-ChIP-Seq(GSE69539)/Homer	-1213	1151	9.17%	517.3	1.43%	6.41
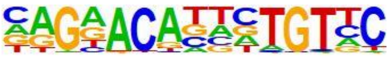	GRE(NR),IR3/RAW264.7-GRE-ChIP-Seq(Unpublished)/Homer	-1359	1319	10.51%	613	1.69%	6.22
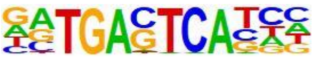	Jun-AP1(bZIP)/K562-cJun-ChIP-Seq(GSE31477)/Homer	-929	944	7.52%	457.9	1.26%	5.97
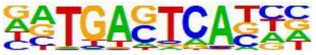	Fosl2(bZIP)/3T3L1-Fosl2-ChIP-Seq(GSE56872)/Homer	-1045	1201	9.57%	672	1.86%	5.15
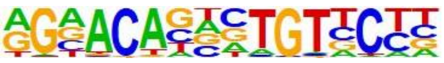	ARE(NR)/LNCAP-AR-ChIP-Seq(GSE27824)/Homer	-1073	1261	10.04%	724.2	2.00%	5.02
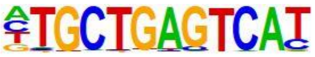	Nrf2(bZIP)/Lymphoblast-Nrf2-ChIP-Seq(GSE37589)/Homer	-111	139	1.11%	84.2	0.23%	4.83

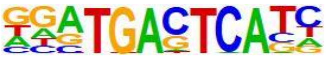	Fra2(bZIP)/Striatum-Fra2-ChIP-Seq(GSE43429)/Homer	-1233	1526	12.16%	931.8	2.57%	4.73
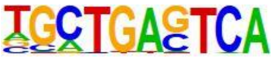	Bach2(bZIP)/OCILy7-Bach2-ChIP-Seq(GSE44420)/Homer	-466	604	4.81%	376.9	1.04%	4.63
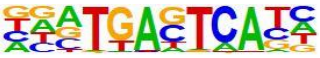	Fra1(bZIP)/BT549-Fra1-ChIP-Seq(GSE46166)/Homer	-1350	1703	13.57%	1065.3	2.94%	4.62
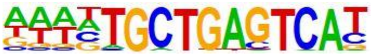	Bach1(bZIP)/K562-Bach1-ChIP-Seq(GSE31477)/Homer	-117	151	1.20%	94.1	0.26%	4.62
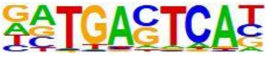	JunB(bZIP)/DendriticCells-Junb-ChIP-Seq(GSE36099)/Homer	-1283	1682	13.40%	1091.4	3.01%	4.45
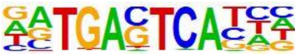	Atf3(bZIP)/GBM-ATF3-ChIP-Seq(GSE33912)/Homer	-1377	1893	15.08%	1293.5	3.57%	4.22
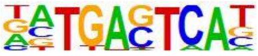	BATF(bZIP)/Th17-BATF-ChIP-Seq(GSE39756)/Homer	-1338	1854	14.77%	1274.5	3.52%	4.20
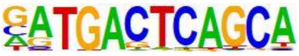	NF-E2(bZIP)/K562-NFE2-ChIP-Seq(GSE31477)/Homer	-111	161	1.28%	112.4	0.31%	4.13
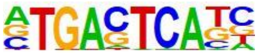	AP-1(bZIP)/ThioMac-PU.1-ChIP-Seq(GSE21512)/Homer	-1254	1978	15.76%	1528.5	4.22%	3.73

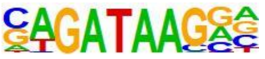	Gata1(Zf)/K562-GATA1-ChIP-Seq(GSE18829)/Homer	-595	1236	9.85%	1150.7	3.18%	3.10
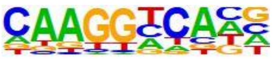	SF1(NR)/H295R-Nr5a1-ChIP-Seq(GSE44220)/Homer	-713	1488	11.85%	1399	3.86%	3.07
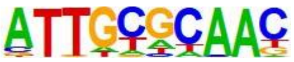	CEBP(bZIP)/ThioMac-CEBPb-ChIP-Seq(GSE21512)/Homer	-631	1326	10.56%	1247.7	3.44%	3.07
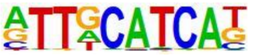	Chop(bZIP)/MEF-Chop-ChIP-Seq(GSE35681)/Homer	-182	401	3.19%	382.7	1.06%	3.01
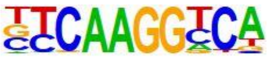	Nr5a2(NR)/mES-Nr5a2-ChIP-Seq(GSE19019)/Homer	-800	1709	13.61%	1641.6	4.53%	3.00
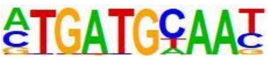	Atf4(bZIP)/MEF-Atf4-ChIP-Seq(GSE35681)/Homer	-251	558	4.44%	536.6	1.48%	3.00
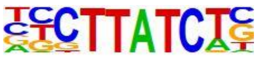	Gata2(Zf)/K562-GATA2-ChIP-Seq(GSE18829)/Homer	-602	1318	10.50%	1274.6	3.52%	2.98
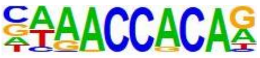	RUNX(Runt)/HPC7-Runx1-ChIP-Seq(GSE22178)/Homer	-854	1988	15.84%	2035.2	5.62%	2.82
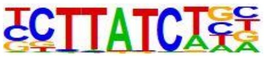	Gata6(Zf)/HUG1N-GATA6-ChIP-Seq(GSE51936)/Homer	-653	1639	13.06%	1740.4	4.80%	2.72
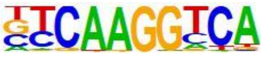	Nr5a2(NR)/Pancreas-LRH1-ChIP-Seq(GSE34295)/Homer	-795	2041	16.26%	2220.4	6.13%	2.65

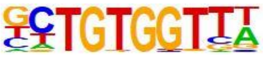	RUNX-AML(Runt)/CD4+-PolII-ChIP-Seq(Barski_et_al.)/Homer	-666	1776	14.15%	1962.4	5.42%	2.61
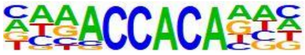	RUNX2(Runt)/PCa-RUNX2-ChIP-Seq(GSE33889)/Homer	-722	1970	15.69%	2216	6.12%	2.56
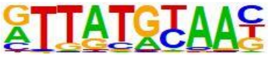	HLF(bZIP)/HSC-HLF.Flag-ChIP-Seq(GSE69817)/Homer	-463	1315	10.47%	1488.1	4.11%	2.55
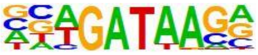	Gata4(Zf)/Heart-Gata4-ChIP-Seq(GSE35151)/Homer	-621	1744	13.89%	1982.2	5.47%	2.54
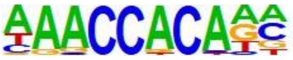	RUNX1(Runt)/Jurkat-RUNX1-ChIP-Seq(GSE29180)/Homer	-839	2361	18.81%	2729.7	7.54%	2.49
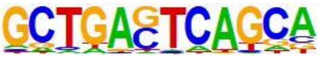	MafK(bZIP)/C2C12-MafK-ChIP-Seq(GSE36030)/Homer	-120	426	3.39%	535.8	1.48%	2.29
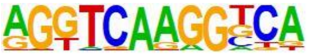	RARg(NR)/ES-RARg-ChIP-Seq(GSE30538)/Homer	-21	73	0.58%	95.8	0.26%	2.23
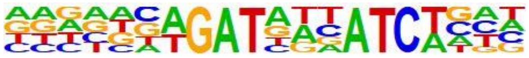	GATA3(Zf)/iTreg-Gata3-ChIP-Seq(GSE20898)/Homer	-600	2244	17.87%	2980.9	8.23%	2.17
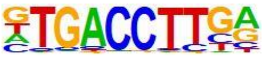	Esrrb(NR)/mES-Esrrb-ChIP-Seq(GSE11431)/Homer	-369	1438	11.45%	1911.8	5.28%	2.17

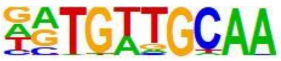	CEBP:AP1(bZIP)/ThioMac-CEBPb-ChIP-Seq(GSE21512)/Homer	-270	1135	9.04%	1550.6	4.28%	2.11
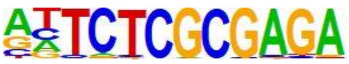	GFX(?)/Promoter/Homer	-22	89	0.71%	122.7	0.34%	2.09
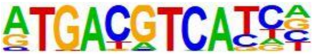	JunD(bZIP)/K562-JunD-ChIP-Seq/Homer	-59	264	2.10%	370	1.02%	2.06
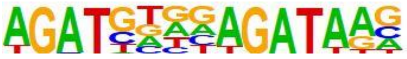	GATA3(Zf),DR4/iTreg-Gata3-ChIP-Seq(GSE20898)/Homer	-17	71	0.57%	101.9	0.28%	2.04

Supplementary data 8: GO analysis of PGR-dependent genes in granulosa cells. Genes were identified from microarrays performed on PRKO vs PGR+/- granulosa cells (5 biological replicates each) and subjected to GO analysis using the *limma* Bioconductor package in R with mm10 terminology and a FDR = 0.05 cut-off. The ten most significantly enriched GO terms from each GO category were selected as representation.

	GO ID	Term	Total gene in pathway	Gene in DEG	p-value
Biological Process	GO:0008152	Metabolic process	10301	9290	0
	GO:0009987	Cellular process	15212	13586	0
	GO:0044237	Cellular metabolic process	9150	8434	0
	GO:0044238	Primary metabolic process	9301	8465	0
	GO:0044260	Cellular macromolecule metabolic process	7374	6781	0
	GO:0044699	Single-organism process	13406	12077	0
	GO:0050789	Regulation of biological process	11067	10008	0
	GO:0050794	Regulation of cellular process	10457	9482	0
	GO:0065007	Biological regulation	11579	10477	0
	GO:0071704	Organic substance metabolic process	9785	8815	0
Molecular Function	GO:0005488	Binding	12634	11636	0
	GO:0005515	Protein binding	8341	7690	0
	GO:0043167	Ion binding	5243	5015	0
	GO:1901363	Heterocyclic compound binding	5189	4845	1.98E-285
	GO:0097159	Organic cyclic compound binding	5284	4923	8.98E-284
	GO:0043169	Cation binding	3460	3310	1.37E-242
	GO:0046872	Metal ion binding	3363	3219	1.07E-236
	GO:0043168	Anion binding	2538	2436	2.74E-180
	GO:0003674	Molecular function	22842	17855	3.34E-157
	GO:0036094	Small molecule binding	2285	2187	1.32E-155
Cellular Component	GO:0005622	Intracellular	13157	12066	0
	GO:0005623	Cell	15313	13931	0
	GO:0005737	Cytoplasm	10208	9534	0
	GO:0044424	Intracellular part	13050	11970	0
	GO:0044444	Cytoplasmic part	7645	7138	0
	GO:0044464	Cell part	15305	13928	0
	GO:0016020	Membrane	9433	8693	0
	GO:0016021	Integral component of membrane	5696	5329	0
	GO:0031224	Intrinsic component of membrane	5847	5469	0
	GO:0043226	Organelle	12350	11423	0

Supplementary data 9: List of genes determined to be significantly differentially expressed in mouse PRKO vs PGR+/- granulosa cell, oviduct and uterus microarrays.

Supplementary data 9: List of genes determined to be significantly differentially expressed in mouse PRKO vs PGR+/- granulosa cell, oviduct and uterus microarrays.

DEG from PRKO vs PGR+/- mouse granulosa cells

Probeset ID	Gene Symbol	RefSeq	p-value	Fold-Change(KO vs. +/-)
10593225	Zbtb16	NM_001033324	0.00032	-13.7345
10498024	Slc7a11	NM_011990	0.00009	-7.5591
10531919	Hsd17b11	NM_053262	0.00009	-6.5212
10380285	Tmem100	NM_026433	0.00277	-5.3345
10573626	Gpt2	NM_173866	0.00010	-4.7675
10381474	Arl4d	NM_025404	0.00254	-4.3798
10493873	Sprr2g	NR_003548	0.00059	-4.0126
10454198	Rnf125	NM_026301	0.00173	-3.7550
10576911	Efnb2	NM_010111	0.00049	-3.7175
10588263	Slco2a1	NM_033314	0.00991	-3.6604
10573979	Gnao1	NM_010308	0.00282	-3.6084
10377215	Gas7	NM_008088	0.00083	-3.5699
10354168	Tbc1d8	NM_018775	0.00035	-3.5541
10500021	Cgn	NM_001037711	0.00136	-3.5274
10568873	Adam8	NM_007403	0.00010	-3.4781
10357472	Cxcr4	NM_009911	0.00032	-3.4756
10494043	Tdrkh	NM_028307	0.00049	-3.4170
10476512	Snap25	NM_011428	0.00009	-3.2078
10463070	Entpd1	NM_009848	0.00008	-3.1707
10506301	Lepr	NM_001122899	0.00915	-3.0562
10498076	Maml3	NM_001004176	0.00282	-3.0398
10438769	Cldn1	NM_016674	0.00173	-3.0150
10527870	Kl	NM_013823	0.00097	-2.8830
10350753	Glul	NM_008131	0.00025	-2.8799
10574023	Mt2	NM_008630	0.00604	-2.8766
10523674	Nudt9	NM_028794	0.00616	-2.8589
10457323	Mkx	NM_177595	0.00173	-2.8130
10382802	Sphk1	NM_011451	0.00019	-2.7980
10488322	A230067G21Rik	NM_001033348	0.00144	-2.7570
10554269	Abhd2	NM_018811	0.00049	-2.7182
10374453	Glul	BC015086	0.00008	-2.6955
10606989	Tsc22d3	NM_001077364	0.00064	-2.6920
10352905	Cd34	NM_001111059	0.00014	-2.6770
10581865	Ldhd	NM_027570	0.00346	-2.6442
10440522	Adamts1	NM_009621	0.00193	-2.5762
10581455	Rbm35b	NM_176838	0.00805	-2.5681
10400143	Stxbp6	NM_144552	0.00113	-2.4634
10554693	Stard5	NM_023377	0.00126	-2.4531
10453629	9430020K01Rik	NM_001081963	0.00254	-2.3975
10409162	Susd3	NM_025491	0.00170	-2.3737
10589773	Lba1	BC086653	0.00997	-2.3663
10567043	Rras2	NM_025846	0.00010	-2.3339
10501229	Gstm1	NM_010358	0.00205	-2.3326
10603492	Porcn	NM_016913	0.00193	-2.2888
10506714	Lrp8	NM_053073	0.00877	-2.2629
10575976	Crispld2	NM_030209	0.00098	-2.2351

10361381	Syne1	NM_001079686	0.00552	-2.1899
10520950	Pdlim1	NM_016861	0.00574	-2.1859
10392347	Pitpnc1	NM_145823	0.00409	-2.1853
10411958	Rnf180	NM_027934	0.00361	-2.1572
10369604	Vps26a	NM_133672	0.00075	-2.1556
10464836	Actn3	NM_013456	0.00115	-2.1455
10534889	Hrbl	NM_178162	0.00409	-2.1182
10539517	Dysf	NM_021469	0.00512	-2.0700
10527936	Fzd1	NM_021457	0.00056	-2.0453
10430179	Apol7b	NM_001024848	0.00161	-2.0430
10405822	Ccrk	NM_053180	0.00026	-2.0308
10469581	Etl4	NM_001081006	0.00891	-2.0301
10374366	Egfr	NM_207655	0.00203	-2.0191
10500837	Dclre1b	NM_133865	0.00272	-2.0147
10392440	Slc16a6	NM_001029842	0.00186	-2.0131
10425040	Apol7b	NM_001024848	0.00136	-2.0047
10448081	Rgmb	NM_178615	0.00272	2.1630

DEG from PRKO vs PGR+/- mouse oviduct

Probeset ID	Gene Symbol	RefSeq	p-value	Fold-Change(KO vs. +/-)
10480090	Itga8	NM_001001309	1.52E-06	-9.866
10494643	Hmgcs2	NM_008256	3.06E-04	-5.812
10603746	Maob	NM_172778	3.37E-04	-5.481
10353252	Gm106	BC147195	1.46E-03	-4.506
10593225	Zbtb16	NM_001033324	5.69E-04	-4.326
10576235	Dpep1	NM_007876	4.73E-03	-4.019
10453057	Cyp1b1	NM_009994	5.85E-03	-3.877
10419854	Slc7a8	NM_016972	9.12E-03	-3.765
10358389	Rgs2	NM_009061	5.78E-04	-3.644
10520633	Tcf23	NM_053085	9.41E-04	-3.564
10347583	Des	NM_010043	3.37E-04	-3.548
10381474	Arl4d	NM_025404	1.79E-03	-3.52
10479165	Edn3	NM_007903	1.15E-03	-3.431
10423049	Prlr	BC096586	1.61E-03	-3.388
10483439	Lrp2	NM_001081088	6.02E-04	-3.316
10440522	Adamts1	NM_009621	7.65E-04	-3.18
10549102	Kcnj8	NM_008428	5.72E-04	-3.149
10599348	Gria3	NM_016886	3.32E-03	-3.145
10568536	Cpxm2	NM_018867	4.92E-04	-3.117
10506488	Ppap2b	NM_080555	4.47E-04	-2.895
10492021	Postn	NM_015784	1.04E-03	-2.865
10547206	Fxyd4	NM_033648	5.46E-04	-2.85
10510574	Errfi1	NM_133753	4.47E-04	-2.779
10568001	Sult1a1	NM_133670	4.47E-04	-2.708
10448836	Tmem204	NM_001001183	4.65E-03	-2.707
10449452	Fkbp5	NM_010220	4.53E-03	-2.683
10595211	Col12a1	NM_007730	9.92E-04	-2.667
10392522	Abca8a	NM_153145	7.29E-03	-2.66
10495449	Col11a1	NM_007729	6.27E-05	-2.642
10511363	Penk1	NM_001002927	5.72E-04	-2.64
10603099	Figf	NM_010216	2.04E-03	-2.629

10572928	Rasd2	ENSMUST0000004	4.93E-03	-2.533
10545707	Actg2	NM_009610	6.27E-05	-2.522
10540028	Klf15	NM_023184	9.60E-04	-2.508
10560329	Hif3a	NM_016868	7.65E-04	-2.505
10466712	Mamdc2	NM_174857	9.10E-04	-2.488
10431410	Mapk11	NM_011161	7.65E-04	-2.47
10394068	Sectm1a	NM_145373	3.12E-03	-2.464
10571601	Pdlim3	NM_016798	1.81E-03	-2.426
10468113	Kcnip2	NM_145703	3.22E-03	-2.418
10489444	Wfdc15b	NM_138685	1.69E-03	-2.396
10423654	Osr2	NM_054049	6.82E-03	-2.38
10583179	Pgr	NM_008829	9.92E-04	-2.374
10413813	Galntl2	NM_030166	9.41E-04	-2.362
10495794	Pde5a	NM_153422	5.69E-04	-2.348
10522467	Rasl11b	NM_026878	3.38E-03	-2.338
10575917	Wfdc1	NM_023395	1.55E-03	-2.323
10540059	Slc41a3	NM_027868	7.65E-04	-2.31
10502805	Ptgfr	NM_008966	1.32E-02	-2.279
10435388	Adcy5	NM_001012765	3.37E-04	-2.272
10386996	Myocd	NM_145136	2.86E-03	-2.267
10361828	Cited2	NM_010828	1.26E-02	-2.258
10417212	Itgbl1	NM_145467	3.37E-04	-2.204
10546432	Adamts9	NM_175314	1.38E-02	-2.195
10363415	Spock2	NM_052994	4.47E-04	-2.19
10501903	Synpo2	BC158045	1.02E-02	-2.188
10362201	Ctgf	NM_010217	1.50E-03	-2.182
10344990	Crispld1	NM_031402	7.65E-04	-2.177
10446473	Lama1	NM_008480	1.48E-02	-2.166
10475643	Fgf7	NM_008008	8.75E-03	-2.152
10405587	Tgfbi	NM_009369	4.73E-04	-2.133
10526241	Cldn3	NM_009902	9.28E-03	-2.128
10544756	Hoxa3	NM_010452	5.11E-03	-2.128
10440534	Adamts5	NM_011782	9.42E-03	-2.117
10607124	Chrdl1	NM_001114385	1.70E-03	-2.109
10405047	Aspn	NM_025711	1.63E-02	-2.093
10544774	Hoxa6	NM_010454	1.67E-02	-2.088
10571142	Gpr124	NM_054044	2.12E-03	-2.087
10387743	Slc2a4	NM_009204	3.58E-03	-2.063
10466800	Pgm5	NM_175013	8.55E-04	-2.05
10354588	Stk17b	NM_133810	8.04E-03	-2.045
10532085	Tgfbr3	NM_011578	4.73E-04	-2.036
10540034	Aldh1l1	NM_027406	1.85E-03	-2.031
10365482	Timp3	NM_011595	5.76E-03	-2.029
10569429	Cdkn1c	NM_009876	9.47E-04	-2.026
10427436	C7	XM_356827	7.54E-03	-2.024
10344981	Pi15	NM_053191	9.35E-04	-2.014
10363157	Pln	NM_023129	3.07E-03	2.033
10517791	Padi4	NM_011061	6.83E-03	2.147
10599001	Agtr2	NM_007429	2.60E-03	2.183
10399854	Slc26a4	NM_011867	5.46E-04	2.685

DEG from PRKO vs PGR+/+ mouse uterus

Probeset ID	Gene Symbol	RefSeq	p-value	Fold-Change (KO vs +/-)
98320_at	Cyp26a1	NM_007811	0.0001768	-9.64
103377_at	Lrp2	NM_001081088	0.0004144	-5.50
94330_at	Npl	NM_028749	0.0003874	-5.28
100539_at	Acot7	NM_133348	0.0001134	-4.88
93328_at	Hdc	NM_008230	0.0002028	-4.83
100046_at	Mthfd2	NM_008638	0.0006155	-4.40
102955_at	Nfil3	NM_017373	0.0034485	-3.49
102430_at	Myd88	NM_010851	0.000993	-3.13
161034_at	Pla2g10	NM_011987	0.0003349	-2.92
94995_at	Ggct	NM_026637	9.91E-05	-2.83
92810_at	Pdk3	NM_145630	0.0043919	-2.76
97890_at	Sgk1	NM_011361	4.33E-05	-2.73
92208_at	Caprin2	NM_181541	6.94E-05	-2.53
96935_at	Pdzk1ip1	NM_026018	0.0003745	-2.52
95597_at	Ptgs1	NM_008969	0.0023747	-2.49
103490_at	Wnt11	NM_009519	0.0052014	-2.43
92796_at	Alpl	NM_007431	0.0098392	-2.43
98792_at	Slc25a48	NM_177809	5.31E-05	-2.37
101973_at	Cited2	NM_010828	0.0027567	-2.31
93270_at	Gars	NM_180678	0.0070477	-2.28
101913_at	Clcn5	NM_016691	0.005943	-2.25
160127_at	Ccng1	NM_009831	0.0001139	-2.24
104174_at	Enpp1	NM_008813	0.0026194	-2.24
160236_at	Slain1	NM_198014	0.0084292	-2.22
96912_s_at	Ctla2a	NM_007796	0.0020732	-2.17
160393_at	Etnk1	NM_029250	0.0036843	-2.05
101561_at	Mt2	NM_008630	0.0075874	-2.04
103514_at	Tnfrsf21	NM_178589	0.0006605	-2.02
160921_at	Acss1	NM_080575	0.0040511	-2.01
103451_at	Ptk2b	NM_172498	0.002168	-2.01
93985_at	Tiparp	NM_178892	0.0033437	-2.00
160565_at	Ckmt1	NM_009897	0.0092232	-2.00
103573_at	Pip5k1b	NM_008846	0.0026046	-2.00
93515_at	Cdh16	NM_007663	0.0089504	2.02
92487_at	Sox7	NM_011446	0.0004988	2.04
101560_at	Emb	NM_010330	0.0008491	2.07
103048_at	Mycn	NM_008709	0.0066011	2.10
102773_at	Car8	NM_007592	0.0075001	2.12
160857_at	Efnb2	NM_010111	0.0005066	2.18
92403_at	St6galnac5	NM_012028	0.0028221	2.26
98423_at	Gjb2	NM_008125	0.0038506	2.89

า

Supplementary data 10: GO analysis of DEG in the uterus, oviduct and granulosa cells. DEG were selected from independent microarrays performed on PRKO vs PGR+/± uterus, oviduct and granulosa cells and subjected to GO analysis using the *limma* Bioconductor package in R with mm10 terminology and a FDR = 0.05 cut-off. For each tissue, the five most significantly enriched GO terms from each GO category were selected as representation.

		GO ID	Term	Total gene in pathway	Gene in DEG	p-value
Biological Process	Uterus	GO:0008219	Cell death	1910	91	5.59927E-17
		GO:0044763	Single-organism cellular process	9986	269	1.64785E-15
		GO:0044699	Single-organism process	13406	330	2.84373E-15
		GO:0044237	Cellular metabolic process	9150	251	6.83939E-15
		GO:0051179	Localisation	5612	174	1.13035E-13
	Granulosa	GO:0044699	Single-organism process	13406	47	7.13258E-07
		GO:2000026	Regulation of multicellular organismal development	1869	16	1.94244E-06
		GO:0050793	Regulation of developmental process	2406	18	2.53583E-06
		GO:0009653	Anatomical structure morphogenesis	2480	18	3.90994E-06
		GO:0044763	Single-organism cellular process	9986	39	4.24759E-06
	Oviduct	GO:0009888	Tissue development	1780	24	3.73383E-10
		GO:0048513	Animal organ development	3233	31	2.04069E-09
		GO:0044767	Single-organism developmental process	5844	42	3.59358E-09
		GO:0032502	Developmental process	5900	42	4.87343E-09
		GO:0072359	Circulatory system development	1015	17	1.04433E-08
Molecular Function	Uterus	GO:0005488	Binding	12634	331	1.08059E-20
		GO:0005515	Protein binding	8341	245	2.39469E-18
		GO:0042802	Identical protein binding	1477	64	2.61609E-10
		GO:0003824	Catalytic activity	5604	159	3.05612E-09
		GO:0097159	Organic cyclic compound binding	5284	150	1.11796E-08
	Granulosa	GO:0005515	Protein binding	8341	39	1.79629E-08
		GO:0005488	Binding	12634	46	3.83712E-07
		GO:0043262	Adenosine-diphosphatase activity	5	2	4.89076E-05
		GO:0042802	Identical protein binding	1477	12	8.17863E-05
		GO:0051015	Actin filament binding	146	4	0.000321108
	Oviduct	GO:0005488	Binding	12634	62	3.76218E-08
		GO:0005539	Glycosaminoglycan binding	181	8	9.40685E-08
		GO:0043167	Ion binding	5243	35	1.3889E-06
		GO:0005515	Protein binding	8341	46	1.94658E-06
		GO:0097367	Carbohydrate derivative binding	2083	20	3.70917E-06
Cellular Component	Uterus	GO:0044464	Cell part	15305	377	5.25646E-22
		GO:0005623	Cell	15313	377	6.00872E-22
		GO:0005737	Cytoplasm	10208	281	3.13185E-18
		GO:0043227	Membrane-bounded organelle	11436	302	1.2542E-17
		GO:0044424	Intracellular part	13050	330	1.73052E-17
	Granulosa	GO:0044464	Cell part	15305	52	1.02037E-08
		GO:0005623	Cell	15313	52	1.04673E-08
		GO:0005886	Plasma membrane	4724	29	2.48526E-08
		GO:0071944	Cell periphery	4841	29	4.38558E-08
		GO:0005622	Intracellular	13157	45	7.75588E-06
	Oviduct	GO:0005578	Proteinaceous extracellular matrix	345	14	2.91366E-12
		GO:0031012	Extracellular matrix	500	15	3.28289E-11
		GO:0005576	Extracellular region	4488	37	1.54077E-09
		GO:0044421	Extracellular region part	3956	34	3.87552E-09
		GO:0071944	Cell periphery	4841	32	6.84718E-06

Supplementary data 11: Canonical pathway analysis of DEG in the uterus, oviduct and granulosa cells. DEG were selected from independent microarrays performed on PRKO vs PGR+/± uterus, oviduct and granulosa cells and subjected to analysis using IPA software with core analysis on default setting. Pathways were selected for those with -log(p-value) ≥ 2 in at least one tissue. -log(p-value) is shown for each tissue type – uterus (dark blue), oviduct (azure), granulosa cells (sky blue). When a pathway is not identified in the tissue, the -log(p-value) bar is missing.

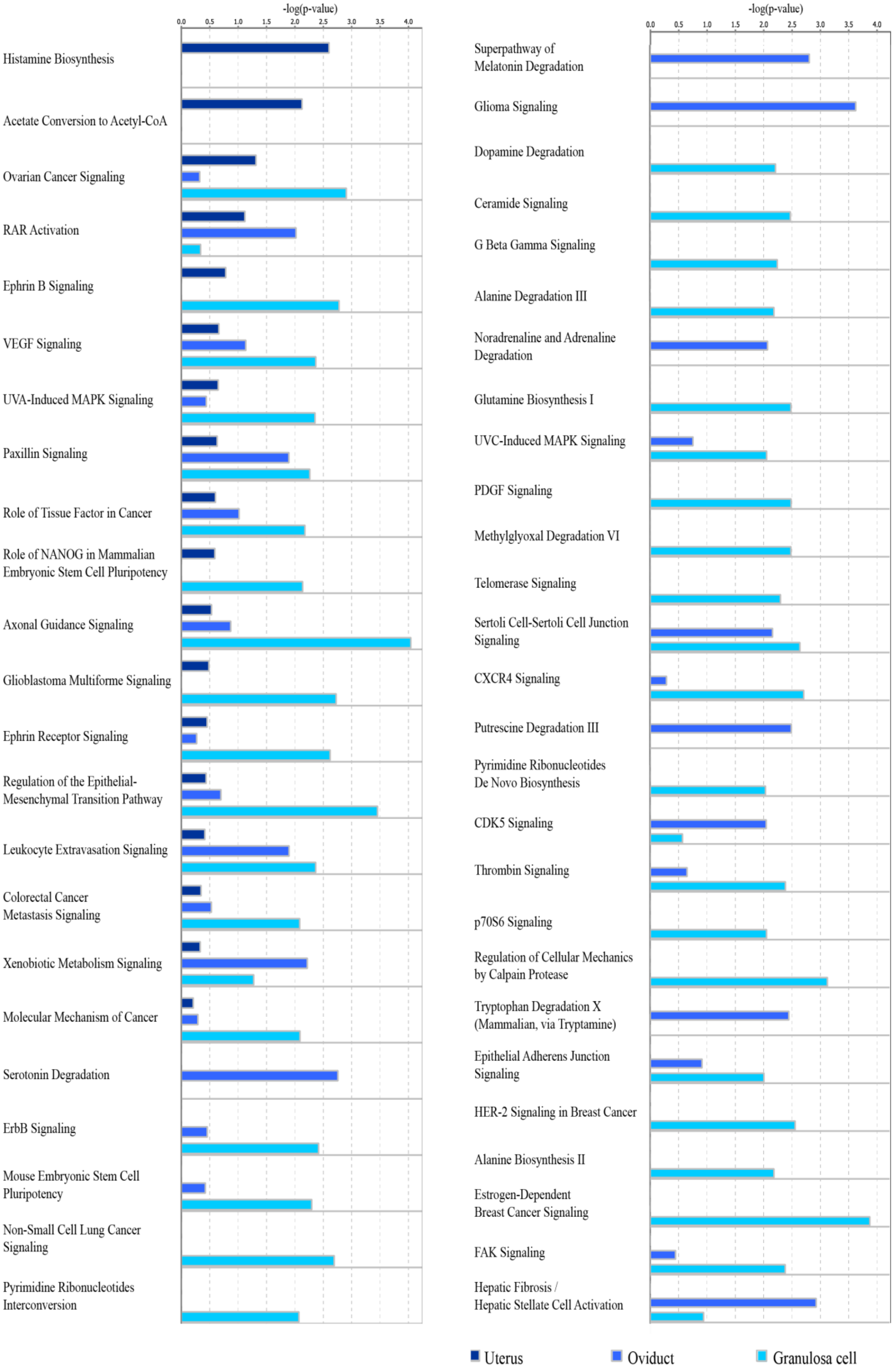

-log(p-value)
0.0 0.5 1.0 1.5 2.0 2.5 3.0 3.5 4.0
Histamine Biosynthesis
Acetate Conversion to Acetyl-CoA
Ovarian Cancer Signaling
RAR Activation
Ephrin B Signaling
VEGF Signaling
UVA-Induced MAPK Signaling
Paxillin Signaling
Role of Tissue Factor in Cancer
Role of NANOG in Mammalian Embryonic Stem Cell Pluripotency
Axonal Guidance Signaling
Glioblastoma Multiforme Signaling
Ephrin Receptor Signaling
Regulation of the Epithelial-Mesenchymal Transition Pathway
Leukocyte Extravasation Signaling
Colorectal Cancer Metastasis Signaling
Xenobiotic Metabolism Signaling
Molecular Mechanism of Cancer
Serotonin Degradation
ErbB Signaling
Mouse Embryonic Stem Cell Pluripotency
Non-Small Cell Lung Cancer Signaling
Pyrimidine Ribonucleotides Interconversion
-log(p-value)
0.0 0.5 1.0 1.5 2.0 2.5 3.0 3.5 4.0
Superpathway of Melatonin Degradation
Glioma Signaling
Dopamine Degradation
Ceramide Signaling
G Beta Gamma Signaling
Alanine Degradation III
Noradrenaline and Adrenaline Degradation
Glutamine Biosynthesis I
UVC-Induced MAPK Signaling
PDGF Signaling
Methylglyoxal Degradation VI
Telomerase Signaling
Sertoli Cell-Sertoli Cell Junction Signaling
CXCR4 Signaling
Putrescine Degradation III
Pyrimidine Ribonucleotides De Novo Biosynthesis
CDK5 Signaling
Thrombin Signaling
p70S6 Signaling
Regulation of Cellular Mechanics by Calpain Protease
Tryptophan Degradation X (Mammalian, via Tryptamine)
Epithelial Adherens Junction Signaling
HER-2 Signaling in Breast Cancer
Alanine Biosynthesis II
Estrogen-Dependent Breast Cancer Signaling
FAK Signaling
Hepatic Fibrosis / Hepatic Stellate Cell Activation
Uterus
Oviduct
Granulosa cell
