# Supplementary material for: Tissue-specific progesterone receptor-chromatin binding and the regulation of progesterone-dependent gene expression
Source: Sci Rep. 2019 Aug 19;9:11966. doi: 10.1038/s41598-019-48333-8 (PMC6700090; doi:10.1038/s41598-019-48333-8)
Supplement: Supplementary file 2 — Supplementary Data 2 [file 41598_2019_48333_MOESM2_ESM.pdf]

```
---
title: "Bioinformatics workflow"
author: "Thao Dinh"
date: "15 June 2018"
output:
  pdf_document: default
  html_document: default
---

```{r setup, include=FALSE}
knitr::opts_chunk$set(echo = TRUE)
```
```

This workflow is contained as supplementary material for the journal article:

Tissue-specific progesterone receptor-chromatin binding and the regulation of progesterone-dependent gene expression

Dinh DT (1), Breen J (1,2), Akison LK (3), DeMayo FJ (4), Brown HM (1,5,6), Robker RL (1) & Russell DL (1)

- (1) Robinson Research Institute, Adelaide Medical School, University of Adelaide, Australia
- (2) University of Adelaide Bioinformatics Hub, University of Adelaide, Australia
- (3) Child Health Research Centre, Centre for Children's Health Research, The University of Queensland, South Brisbane, Qld 4101, Australia
- (4) Pregnancy and Female Reproduction Group, National Institute of Environmental Health Sciences, Research Triangle Park, NC 27709
- (5) South Australian Health and Medical Research Institute, Adelaide, South Australia, Australia
- (6) Australian Research Council Centre for Nanoscale Biophotonics, University of Adelaide, Australia

Introduction

Progesterone receptor (PGR) co-ordinately regulates ovulation, fertilisation and embryo implantation through tissue-specific actions, but the mechanisms for divergent PGR action are poorly understood. Here we characterised PGR activity in mouse granulosa cells using combined ChIP-seq for PGR and H3K27ac and compared PGR properties in granulosa cells versus the uterus. This is the first genome-wide description of PGR action in granulosa cells and systematic comparison of diverse PGR action in different reproductive tissues. These results clarify finely-tuned contextual PGR-chromatin interactions with implications for more targeted reproductive medicine.

Workflow

This analysis was carried out in both command-line bash and R, primarily with the use of the R package `systemPipeR`. The general workflow is as

follows:
1. Download datasets from NCBI SRA or raw data in FASTQ file obtained from sequencing
2. Quality control using FastQC
3. Obtain and index mouse genome mm10
4. Run alignments with bowtie2
5. Call peaks with MACS2
6. Obtain correctly edited XLS peak files and convert object type to fit package requirements
7. Plot read count frequency
8. Plot genome distribution and obtain gene annotation list
9. Identify and plot peak overlapping between datasets
10. Common motif analysis with HOMER
11. Visualise data on UCSC Genome Browser

Specialised packages required for ChIP-seq analysis.

These can be installed using biocLite or BiocManager::install() functions.

```{r}
library(ChIPseeker)
library(TxDb.Mmusculus.UCSC.mm10.ensGene)
library(BSgenome.Mmusculus.UCSC.mm10)
library(GenomicRanges)
library(tidyverse)
library(rtracklayer)
library(Rcade)
library(ChIPpeakAnno)
library(clusterProfiler)
library(BayesPeak)
library(VennDiagram)
library(readxl)
library(org.Mm.eg.db)

```

GRANULOSA CELL data

Granulosa cell data is received as compressed FASTQ file, obtained via FPT from Active Motif. There are four files in total, for PGR replicate 1, PGR replicate 2, H3K27ac and pooled input, renamed as:
./PGR_rep1.fastq.gz
./PGR_rep2.fastq.gz
./H3K27ac.fastq.gz
./pooled_input.fast.gz

```{bash}

# Unzip files
gunzip ./PGR_rep1.fastq.fa
gunzip ./PGR_rep1.fastq.fa
gunzip ./PGR_rep1.fastq.fa
gunzip ./PGR_rep1.fastq.fa
```

````
```

## Make parameter files for quality check

Create two text files as follow:
1. Parameter:
./bowtieSE.param is a text file with the following content:

```
## Parameter file for Bowtie2 SE
## Values in <...> reference column titles in targets file

PairSet Name Value
modules NA bowtie2/2.2.5
software NA bowtie2
cores -p 16
other NA -k 50 --non-deterministic
reference -x ./mm10
infile1 -U
infile1 path NA
infile2 NA
infile2 path NA
outfile1 -S
outfile1 path ./
outfile1 remove NA
outfile1 append .sam
outfile1 outextension .bam
```

2. Target:
./ChIP_targets.txt is a text file with the following content:

```
FileName SampleName Factor SampleLong
./PGR_rep1.fastq PR1 PR rep1-2_PR_mm_i80
./PGR_rep2.fastq PR2 PR rep4_PR_mm_i81
./H3K27ac.fastq H3K27ac H3K27ac rep4_H3K27Ac_mm_i79
```


## Check quality of data

```{r}

# Check quality
args <- systemArgs(sysma="./bowtieSE.param",
                   mytargets="./ChIP_targets.txt")
fqlist <- seeFastq(fastq=infile1(args), batchsize=100000, klength=8)

# Plot report
pdf("~/fastqReport.pdf", height=18, width=4*length(fqlist))
seeFastqPlot(fqlist)
dev.off()

```
````
````

Prepare and index mouse genome

Mouse genome mm10 is used and downloaded from the mouse reference base at the Sanger Institute. Before being used for bowtie2, mouse genome is indexed using bowtie2-build.

```{bash}

# Download fasta of mm10
wget ftp://ftp-mouse.sanger.ac.uk/ref/GRCm38_68.fa.gz

# Unzip
gunzip ~/mm10/GRCm38_68.fa

# Build bowtie2 genome index
bowtie2-build ~/mm10/GRCm38_68.fa ~/mm10

# Rezip to save space
gzip ~/mm10/GRCm38_68.fa

```

Alignment to genome with bowtie2

Bowtie2 is used to align sequences to mm10 mouse genome.
Specific parameters are as follow, applied from the authors' previous experiences on ChIP-seq data:
  -p 16 (number of running threads, to increase memory footprint)
  -k 50 (alignment in k mode: search for up to 50 valid alignments for each read)
  --non-deterministic (initiate pseudo-random identification of reads)

```{bash}

# Align data
bowtie2 -p 16 -k 50 --non-deterministic -x ~/mm10 -U ./PGR_rep1.fastq  -S ~/pr1.fastq.sam
bowtie2 -p 16 -k 50 --non-deterministic -x ~/mm10 -U ./PGR_rep2.fastq  -S ~/pr2.fastq.sam
bowtie2 -p 16 -k 50 --non-deterministic -x ~/mm10 -U ./H3K27ac.fastq  -S ~/h3k27ac.fastq.sam
bowtie2 -p 16 -k 50 --non-deterministic -x ~/mm10 -U ./pooled_input.fastq -S ~/input.fastq.sam

```

Bowtie2 output is a SAM file. To make this available for peak calling with MACS2, these need to be converted from SAM to BAM file.

```{bash}

# Convert to bam files (again to save some space)
samtools view -Sb ./pr1.fastq.sam > ./pr1.fastq.bam
```

```
samtools view -Sb ./pr2.fastq.sam > ./pr2.fastq.bam
samtools view -Sb ./h3k27ac.fastq.sam > ./h3k27ac.fastq.bam
samtools view -Sb ./input.fastq.sam > ./input.fastq.bam

```

Call peaks with MACS2

MACS2 setting: mouse genome size (1.87e9) is as indicated in MACS2 manual (https://github.com/taoliu/MACS). p-value cut-off is the same as that used in the original uterus ChIP-seq publication (p cut-off = 10e-10). Input control was used as the baseline to call peaks.

```{bash}

# Call peaks
macs2 callpeak  -t ~/pr1.fastq.bam -c ~/input.fastq.bam \
  -n ~/pr1_vs_input.macs2 -f BAM -g 1.87e9 -B -p 10e-10 --nomodel

macs2 callpeak  -t ~/pr2.fastq.bam -c ~/input.fastq.bam \
  -n ~/pr2_vs_input.macs2 -f BAM -g 1.87e9 -B -p 10e-10 --nomodel

macs2 callpeak  -t ~/h3k27ac.fastq.bam -c ~/input.fastq.bam \
  -n ~/h3k27ac_vs_input.macs2 -f BAM -g 1.87e9 -B -p 10e-10 --nomodel

```

MACS2 output includes the following:
- XLS file (spreadsheet of all called peaks)
- BED file (for UCSC genome browser visualization)
- NARROWPEAK file (for GEO submission)
- 2 BDG file (control_lambda and treat_pileup)

UTERUS data

Uterus data has been described in Rubel, C. A. et al. Molecular Endocrinology 26, 1428-1442, (2012) and is publicly available in GEO database (GEO accession number: GSE34927). Raw data is available as SRA file.

Retrieve raw data:

SRA files are obtained from:
- SRX114724 (P4 treatment)
- SRX114723 (oil treatment); and,
- SRX114726 (input)

```{bash}

# Download and convert using SRA toolkit
wget ftp://ftp-trace.ncbi.nlm.nih.gov/sra/sra-
instant/reads/ByRun/sra/SRR/SRR396/SRR396854/SRR396854.sra
fastq-dump ~/SRR396854.sra -O ~/FASTQ
```

wget ftp://ftp-trace.ncbi.nlm.nih.gov/sra/sra-instant/reads/ByRun/sra/SRR/SRR396/SRR396854/SRR396853.sra
fastq-dump ~/SRR396853.sra -O ~/FASTQ

wget ftp://ftp-trace.ncbi.nlm.nih.gov/sra/sra-instant/reads/ByRun/sra/SRR/SRR396/SRR396854/SRR396856.sra
fastq-dump ~/SRR396856.sra -O ~/FASTQ

```

Once FASTQ files are obtained, the workflow used for granulosa cell data is applied (for alignment and peak calling).

---

# ChIP-seq analysis

The following analysis requires the XLS file from MACS2. This XLS file behaves like a TXT file and thus if opened as it is, column names will be shifted. Thus, before starting analysis, open the XLS file in Excel and adjust the column name to the right data column. Also, to make this file readable in R, save this file as the correct XLS/XLSX format. Rename the file to make it more user-friendly if desired.

## Get mouse genome database

Mouse genome database is from UCSC mm10.

```{r}

# Transcript annotation for the mm10 data
txdb <- TxDb.Mmusculus.UCSC.mm10.ensGene

```

Retrieve XLS file (after column adjustment)

```{r}

# Get data from XLS files
pr1Peaks <- read_excel("./pr1_input.xls")
pr2Peaks <- read_excel("./pr2_input.xls")
h3k27acPeaks <- read_excel("./h3k27ac_input.xls")

```

Make GRanges object

This is a requirement of the `ChIPseeker` package. This object contains the following data:
- chr (chromosomal location of peak)
```

- start (start of peak)
- end (end of peak)
- summit (absolute peak summit position)
- score (pileup height at peak summit, -log10(pvalue) for the peak summit)
- peak (ID of peak)

```{r}

# Make GRanges object
pr1Summit <- pr1Peaks %>%
  dplyr::select(chr = chr, start = start, end = end,
                summit = `abs_summit`,
                score = `Peak Val`,
                peak = name) %>%
  makeGRangesFromDataFrame(keep.extra.columns = TRUE)
pr2Summit <- pr2Peaks %>%
  dplyr::select(chr = chr, start = start, end = end,
                summit = `abs_summit`,
                score = `Peak Val`,
                peak = name) %>%
  makeGRangesFromDataFrame(keep.extra.columns = TRUE)
h3k27acSummit <- h3k27acPeaks %>%
  dplyr::select(chr = chr, start = start, end = end,
                summit = `abs_summit`,
                score = `Peak Val`,
                peak = name) %>%
  makeGRangesFromDataFrame(keep.extra.columns = TRUE)

# Combine GRanges objects into a list for combined analyses afterwards
summits <- list(pr1Summit, pr2Summit, h3k27acSummit)

```

Plot read count frequency

Genomic feature annotation to peaks is done using the getTagMatrix function of `ChIPseeker` package. The genomic range is assigned as 3 kb upstream/downstream of a gene using mouse genome mm10 (UCSC) as database, then peaks are assigned to genome range. The tag matrix object is used to plot read count frequency of peaks, using the plotAvgProf function, with the confidence interval cut-off at 0.95.

```{r}

# Retrieve genomic details, with genomic range assigned as +/-3000bp
promoter <- getPromoters(txdb, upstream=3000, downstream=3000)

# Applied genomic range retrieved above to data
tagMatrixList <- lapply(summits, getTagMatrix, windows=promoter)

# Assign names to datasets
names(tagMatrixList) <- c("pr1", "pr2", "h3k27ac")

# Create read count frequency graph data, limit graph to TSS +/-3000bp on x
axis, confidence interval cut-off = 0.95.
```

```
p <- plotAvgProf(tagMatrixList, xlim=c(-3000, 3000), conf=0.95,resample=500)

# Actual plotting of read count frequency. While the plotAvgProf function alone would still give you the graph, it would not be very aesthetically pleasing, so ggplot was used to make a prettier graph
ggplot(p$data, aes(pos, value, group=.id, color=.id)) +
  geom_line(linetype = 1, size = 1.3) +
  geom_vline(xintercept=0,
             linetype="longdash") +
  scale_x_continuous(breaks=c(-3000, -1500, 0, 1500, 3000), # Section x axis to assigned intervals
                     labels=c(-3000, -1500, 0, 1500, 3000)) + # Label intervals on axis
  scale_y_continuous(breaks=c(0, 0.00025, 0.00050, 0.00075, 0.001, 0.00125), # Section y axis to intervals
                     labels=c(0, 0.00025, 0.00050, 0.00075, 0.001, 0.00125)) +
  ylab("Read Count Frequency") + # Axis labelling
  xlab("Distance from TSS") +
  theme_bw() +
  theme(panel.grid.minor = element_blank(), panel.grid.major = element_blank()) +
  theme(legend.title=element_blank(), legend.position="bottom")

```

Plot peak genome distribution (piechart)

To obtain genome distribution of peaks, the annotatePeak function from `ChIPseeker` package is used, which assign genomic features from database (mouse genome mm10, UCSC) to peaks. Genomic range is set at 3 kb upstream/downstream of a gene. Once assigned, piechart for each dataset is plotted.

```{r}

# Get genomic annotation from data as GRanges object
peakAnnoList <- lapply(summits, annotatePeak, txdb, tssRegion=c(-3000, 3000), verbose=FALSE)

# Assign name to datasets for labelling
names(peakAnnoList) <- c("pr1", "pr2", "h3k27ac")

# Plot individual piechart for the genomic distribution of each dataset
plotAnnoPie(peakAnnoList[[1]]) #PR1
plotAnnoPie(peakAnnoList[[2]]) #PR2
plotAnnoPie(peakAnnoList[[3]]) #H3K27ac

```

Obtain genomic annotation list

The above command will not directly give a list of genomic annotation of peaks. To obtain the actual list of gene annotation for peaks, the

annotatePeak function from `ChIPseeker` package is used as below.

```{r}

# Get genomic annotation from data as GRanges object
peakAnno <- annotatePeak(pr1Summit, tssRegion=c(-3000, 3000),
                         TxDb=txdb, annoDb="org.Mm.eg.db")

#Create data.frame object from annotation result (compatible to be exported
as readable XLS file)
peakAnnoframe <- as.data.frame(peakAnno)

#Export data as XLS file
write.table(peakAnnoframe, file = "~/pr1_gene_list.xls", sep = "\t")

#Repeat the above for PR2 data
peakAnno <- annotatePeak(pr2Summit, tssRegion=c(-3000, 3000),
                         TxDb=txdb, annoDb="org.Mm.eg.db")
peakAnnoframe <- as.data.frame(peakAnno)
write.table(peakAnnoframe, file = "~/pr2_gene_list.xls", sep = "\t")

```

Peak comparison between datasets

In comparing two datasets (eg between uterus vs granulosa PGR binding sites), datasets can be overlapped and unique peaks / overlapped peaks can be extracted for further analyses. Overlapping of peaks is done using the findOverlapsOfPeaks function of `ChIPpeakAnno` package.
The following example is for overlap between PR1 and PR2 dataset.

Retrieve dataset-unique peaks

Dataset-unique peaks can be extracted from the overlap function described above. Dataset-unique peaks are listed in the same file, which can be separated by name.

```{r}

# Find overlapping peaks between two datasets
overlap <- findOverlapsOfPeaks(pr1Summit, pr2Summit, maxgap=100,
connectedPeaks=c("min"))

# Extract data-unique (i.e PR1-unique or PR-2 unique) peaks
unique_peaks <- as.data.frame(overlap$uniquePeaks)

# Filter PR1-unique peaks by name
pr1_unique <- filter(unique_peaks, grepl('pr1', seqnames))

# Export PR1-unique peaks to XLS file
write.table(pr1_unique, file = "~/unique_pr1.xls", sep = "\t")

# Filter PR2-unique peaks by name
pr2_unique <- filter(unique_peaks, grepl('pr2', seqnames))
```

```
# Export PR2-unique peaks to XLS file
write.table(pr2_unique, file = "~/unique_pr2.xls", sep = "\t")
```

Get overlapped peaks

From the same overlap result, overlapping peaks can be extracted. The resulting dataset contains duplicates of peaks, sometimes more than two if a peak from one dataset overlaps more than one peak from the other dataset (happens when a broad peak overlaps multiple narrower peaks). To remove the duplicates (required for `ChIPseeker` analyses), peaks with broader width are filtered out and only narrow peaks are kept (regardless of which dataset the peak belongs to). This ensures that sharper, more precise peaks are retained.

```{r}

# Extract overlapping peaks
intersect <- as.data.frame(overlap$overlappingPeaks)

# Filter overlapping peaks in which the PR1 peak is narrower or of equal
width to the correspondingly overlapped PR2 peak)
pr1_to_pr2 <- filter(intersect, pr1_width <= pr2_width)

# Export overlapped peaks (PR1<=PR2) to XLS file
write.table(pr1_to_pr2, file = "~/intersect_pr1_to_pr2.xls", sep = "\t")

# Filter overlapping peaks in which the PR1 peak is wider than the
correspondingly overlapped PR2 peak)
pr2_to_pr1 <- filter(ut_merged, pr1_width > pr2_width)

# Export overlapped peaks (PR1>PR2) to XLS file
write.table(pr2_to_pr1, file = "~/intersect_pr2_to_pr1.xls", sep = "\t")

```

At the end, open both intersect XLS file and pool together. Misalignment of column name might occur during the above filter step, which can be mitigated by exporting the 'intersect' dataframe above and reassign column names in Excel.

Generate venn diagrams with known size of dataset

(see Environment panel for total row/peak count)

To plot a venn diagram comparing two or more datasets, use the number of overlapped peaks / genes as indicated on the XLS file or the Data panel in the Environment window of RStudio. These numbers will need to be manually added to the script, in the following order:
'Dataset1_unique, Dataset2_unique, overlap' (in the following example, 8158, 5478, 810).

Note that in venn diagrams comparing three or more datasets, the circles

will not be proportionate to the size of dataset and will need to be manually adjusted using a graphic design software.

```{r}

# Plot graph in a new page each time
grid.newpage()

# Plot Venn diagram with two datasets
draw.pairwise.venn(8158, 5478, 810, category = c("Granulosa", "Uterus"),
lwd = rep(1, 1), col = rep("black", 2), fill = c("blue","orange"), alpha =
rep(0.5, 2), cat.pos = c(0, 0), cat.dist = rep(0.05, 2))

# Plot Venn diagram with three datasets
overrideTriple = 1
draw.triple.venn(area1 = 41, area2 = 81, area3 = 61, n12 = 2, n23 = 3, n13
= 2,
n123 = 0, category = c("Uterus", "Oviduct", "Granulosa"), lwd = rep(1, 1),
col = rep("black", 3), fill = c("blue", "orange", "mediumorchid"),
overrideTriple = 2)

```

Motif analysis with HOMER

HOMER (http://homer.ucsd.edu/homer/index.html) is used to identify enriched motifs.
This tool requires BED file which is generated from MACS2. If a custom-made peak file is required (for example, in the case of intersect PGR peaks) then a custom-made BED file can be created by retrieving the following columns from XSL file: chr, start, end, peak_name into a new XLS file. Change the filetype of this file as BED.

```{bash}

# Install the mm10 mouse genome package from HOMER
perl configureHomer.pl -install mm10

# Find known and de novo motifs, with the region size setting at 200bp
(reccommended for finding primary and co-enriched motifs for transcription
factor)
findMotifsGenome.pl pr1.bed mm10 ./HOMER/pr1 -size 200 -mask
findMotifsGenome.pl pr2.bed mm10 ./HOMER/pr2 -size 200 -mask
findMotifsGenome.pl h3k27ac.bed mm10 ./HOMER/h3k27ac -size 200 -mask

```

Visualisation of ChIP-seq data

To visualise ChIP-seq data on UCSC Genome Browser, either BED file can be used, or, more consistently, BIGWIG file can be generated from BDG file (treat_pileup) obtained from MACS2. This can be done using the `wigToBigWig` program (UCSC). This is also available on Galaxy (www.usegalaxy.org) which was used for this example.

The PR1 data is used for the example below. Before proceeding, rename file to something more manageable.

First the BDG file needs to be edited to the correct format.

```{bash}

#Remove peaks aligned to unlocalised contigs ('chromsome' name usually starts with G, M and L). These contigs are not recognised for visualisation in the genome browser and need to be removed.
sed '/^J/d; /^M/d; /^G/d' PR1.bdg > Pr1_filtered.bdg

```

One other criteria required for BIGWIG file conversion and visualisation is that the chromosome annotation needs a 'chr' prefix (eg chrX instead of X). The BDG file generated by MACS does not have the 'chr' prefix and this needs to be added manually.

```{bash}

# Extract first column (chromosome column) of BDG file for editing and place this column in a new TXT file.
awk '{print $1}' PR1_filtered.bdg > PR1_1.txt

# Add the prefix 'chr' to data from the extracted first column and place this in a new TXT file.
awk '$0="chr"$0' PR1_1.txt > PR11.txt

# Remove the first column from the original BDG file.
awk '{$1=""}1' PR1_filtered.bdg | awk '{$1=$1}1' >
PR1_filtered_no_column1.bdg

# Add the edited first column to the BDG file, thereby replacing the old column with the new column.
paste PR11.txt PR1_filtered_no_column1.bdg | column -s $'\t' -t >
PR1_edited.bdg

```

At this point the BDG file can be converted to BIGWIG.
On Galaxy, upload the edited BDG file and use the `Wig/BedGraph-to-bigWig converter` tool at Default settings. The resulting BIGWIG file can be visualised on UCSC using the direct link on Galaxy.
